# Advances in Cellular Immune Theranostic Approaches for Glioblastoma: Current Trends and Future Directions

**DOI:** 10.1002/cai2.70018

**Published:** 2025-07-03

**Authors:** Ying Gong, Wanying Lin, Xuechun Fang, Ruyi Zhang, Min Luo, Haoran Wu, Shuai Chu, Chuangkun Li, Yiming Peng, Zhiyan Piao, Siping Wu, Junhao Li, ZongZhong He, Haixia Li, Hongxia Wang

**Affiliations:** ^1^ Department of Laboratory Medicine, Guangdong Provincial Key Laboratory of Precision Medical Diagnostics, Guangdong Provincial Key Laboratory of Single‐cell and Extracellular Vesicles Guangdong Engineering and Technology Research Center for Rapid Diagnostic Biosensors, Nanfang Hospital Southern Medical University Guangdong Guangzhou China; ^2^ Guangdong Provincial Clinical Research Center for Laboratory Medicine Nanfang Hospital Southern Medical University Guangdong Guangzhou China; ^3^ Department of Internal Medicine, Division of Hematology University of Maastricht Maastricht the Netherlands; ^4^ Department of Transfusion Medicine of General Hospital of Southern Theatre Command Guangdong Guangzhou China

**Keywords:** CAR‐T therapy, checkpoint inhibitor, glioblastoma, immunotherapy, tumor microenvironment

## Abstract

Glioblastoma is a highly malignant type of brain tumor that remains one of the most challenging cancers to treat because of its aggressive nature, genetic heterogeneity, and immunosuppressive tumor microenvironment. Despite advances in standard treatments, such as surgery, radiation, and chemotherapy, patient outcomes remain poor, driving the need for innovative therapeutic approaches. Cellular immune theranostics, which combines therapeutic and diagnostic capabilities, has emerged as a promising strategy to combat glioblastoma. The present review discusses recent advances in cellular immunotherapy, including the development and application of chimeric antigen receptor T cells, chimeric antigen receptor natural killer cells, and macrophage‐based therapies. In addition, this review highlights the potential of oncolytic viruses and personalized tumor vaccines for improving immunotherapy outcomes. The integration of advanced diagnostic tools, such as the real‐time monitoring of therapeutic responses through immunobiomarkers and imaging techniques, is emphasized as crucial for optimizing treatment strategies. However, important challenges remain, including the complexity of immune cell engineering, the difficulties of therapeutic delivery across the blood–brain barrier, and the immunosuppressive properties of the tumor microenvironment. Overcoming these challenges through innovative methodologies will be vital for improving the efficacy of cellular immune theranostics in the treatment of glioblastoma, with the ultimate goal of improving patient survival and quality of life.

## Graphical abstract

Glioblastoma (GBM) remains a highly malignant brain tumor with limited treatment efficacy, driving the need for innovative cellular immune theranostic strategies that integrate diagnostic and therapeutic approaches. Recent advances in cellular immunotherapy for GBM include chimeric antigen receptor (CAR)‐T/NK cells, macrophage‐based therapies, oncolytic viruses, and personalized tumor vaccines, while advanced diagnostic tools like single‐cell RNA sequencing and immune biomarkers enhance treatment monitoring. Key challenges in GBM immunotherapy involve overcoming the blood–brain barrier, addressing immune suppression in the tumor microenvironment, and optimizing the engineering of immune cells for durable clinical responses.

**Abbreviations**
AAV: adeno‐associated viral vector
Akt: protein kinase B
aPD‐1: anti‐PD‐1 immunoadhesin
B7H3: B7 homolog 3
BBB: blood–brain barrier
BBM: blood‐borne myeloids
BTG1: BTG anti‐proliferation factor 1
CaMKII: Ca^2+^/calmodulin‐dependent protein kinase II
CAR: chimeric antigen receptor
CCL5: chemokine C‐C ligand 5
CED: convection‐enhanced delivery
CNS: central nervous system
CRS: cytokine release syndrome
CSF‐1: colony‐stimulating factor 1
CSF‐1R: colony‐stimulating factor 1 receptor
CTLA‐4: cytotoxic T lymphocyte‐associated protein 4
CX3CR1: CX3C motif chemokine receptor 1
CXCR: C‐X‐C chemokine receptor
DC: dendritic cell
EGFRvIII: epidermal growth factor receptor variant III
EVs: extracellular vesicles
GAM: glioma‐associated microglia/macrophages
GBM: glioblastoma multiforme
GM‐CSF: granulocyte–macrophage colony‐stimulating factor
GSCs: GBM stem cells
HER2: receptor protein‐tyrosine kinase
HSV: herpes simplex virus
ICANS: immune effector cell‐associated neurotoxicity syndrome
IFN: interferon
IL: interleukin
iNK: induced pluripotent stem cell‐derived natural killer cells
MDSC: myeloid‐derived suppressor cells
MHC: major histocompatibility complex
MHC‐II: major histocompatibility complex class II
MMP2: matrix metalloproteinase 2
MRI: magnetic resonance imaging
NK: natural killer
NKG2D: natural killer group 2D
NKT: natural killer T cells
NLR: neutrophil‐to‐lymphocyte ratio
NR4A2: nuclear receptor subfamily 4 group A member 2
OV: oncolytic viruses
PD‐1: programmed cell death protein 1
PD‐L1: programmed cell death ligand 1
PET: positron emission tomography
p‐: phosphorylated
RANO: response assessment in neuro‐oncology
TAMs: tumor‐associated macrophages
TGF‐β: transforming growth factor‐beta
Th: T helper
TME: tumor microenvironment
TNF‐α: tumor necrosis factor‐alpha
TTFields: tumor‐treating fields

## 1 Introduction

Glioblastoma multiforme (GBM) originates from the neuroepithelium; it constitutes 40%–50% of all craniocerebral tumors, positioning them as the most prevalent form of intracranial malignant neoplasms [1]. Data from the Central Brain Tumor Registry of the United States indicate that the incidence rate of all primary brain and other central nervous system (CNS) tumors among children and adolescents aged 0–19 years stands at 6.13 per 100,000 people [2]. GBMs are more prevalent in males, whereas meningiomas are more common in females [3]. The defining biological features of GBM cells include their ability to self‐renew and differentiate, coupled with an exceptionally high level of heterogeneity [4]. Various GBM cell types and cells at different stages of development show unique characteristics and manifestations, which can play pivotal roles in the malignant progression of these tumors [5, 6].

Current diagnostic approaches for GBM in clinical practice encompass a multifaceted approach, integrating advanced imaging, histopathological examination, and molecular analysis [7, 8]. Over the past few years, molecular diagnostics have become increasingly important, with assessments of genetic mutations such as *IDH1/2*, *MGMT* promoter methylation, and 1p/19q co‐deletion playing pivotal roles. Together, these diagnostic tools constitute a comprehensive strategy that bolsters the accuracy of GBM diagnosis, enables early intervention, and enhances patient outcomes [4, 9]. Standard treatments for GBM, including surgery, radiation therapy, and chemotherapy, often yield limited success because of the tumor's aggressive nature, genetic diversity, and capacity to evade traditional treatments, leading to frequent recurrence and poor patient prognosis [10]. These challenges emphasize the pressing need for innovative therapeutic and diagnostic approaches. Targeted molecular therapies, immunotherapies, advanced imaging techniques, and personalized medicine strategies that consider the unique genetic and molecular characteristics of each tumor are among the promising areas of research [11]. Theranostics, a fusion of “therapy” and “diagnostics,” represents a pioneering concept in personalized medicine. It integrates diagnostic and therapeutic processes to tailor treatments to individual patients [12]. Within this framework, cellular immune theranostics is emerging as a promising strategy against GBM [13]. This approach combines immune‐based therapies with advanced diagnostic tools, leveraging the immune system to target and eliminate cancer cells while simultaneously using diagnostic techniques to monitor immune responses and tumor dynamics [14]. By offering a potent, personalized approach to treating this aggressive brain cancer, cellular immune theranostics has the potential to improve survival rates and quality of life of patients [15, 16].

The present review aims to provide a comprehensive overview of the latest advances in cellular immune theranostic approaches for GBM, encompassing chimeric antigen receptor (CAR)‐T cells, CAR‐natural killer (NK) cells, macrophage‐based therapies, and oncolytic viruses (OVs). Furthermore, this review emphasizes the integration of advanced diagnostic tools, such as the real‐time monitoring of therapeutic responses through immune biomarkers and imaging techniques, which are essential for refining treatment strategies. Another primary focus of the review is to address the substantial challenges associated with the complexity of immune cell engineering, the delivery of therapies across the blood–brain barrier (BBB), and the immunosuppressive nature of the tumor microenvironment (TME). These challenges are critical barriers that need to be overcome to improve the effectiveness of immune‐based therapies for GBM. By providing a comprehensive overview of the latest advances and addressing these challenges, the current review aims to pave the way for future innovations in the field. These innovations may lead to more effective and personalized treatments for GBM, ultimately improving patient survival and quality of life. The ultimate goal is to harness the full potential of cellular immune theranostics to make a meaningful impact on the treatment of this aggressive brain cancer.

## 2 Cellular Components of GBM

**Figure 1:**
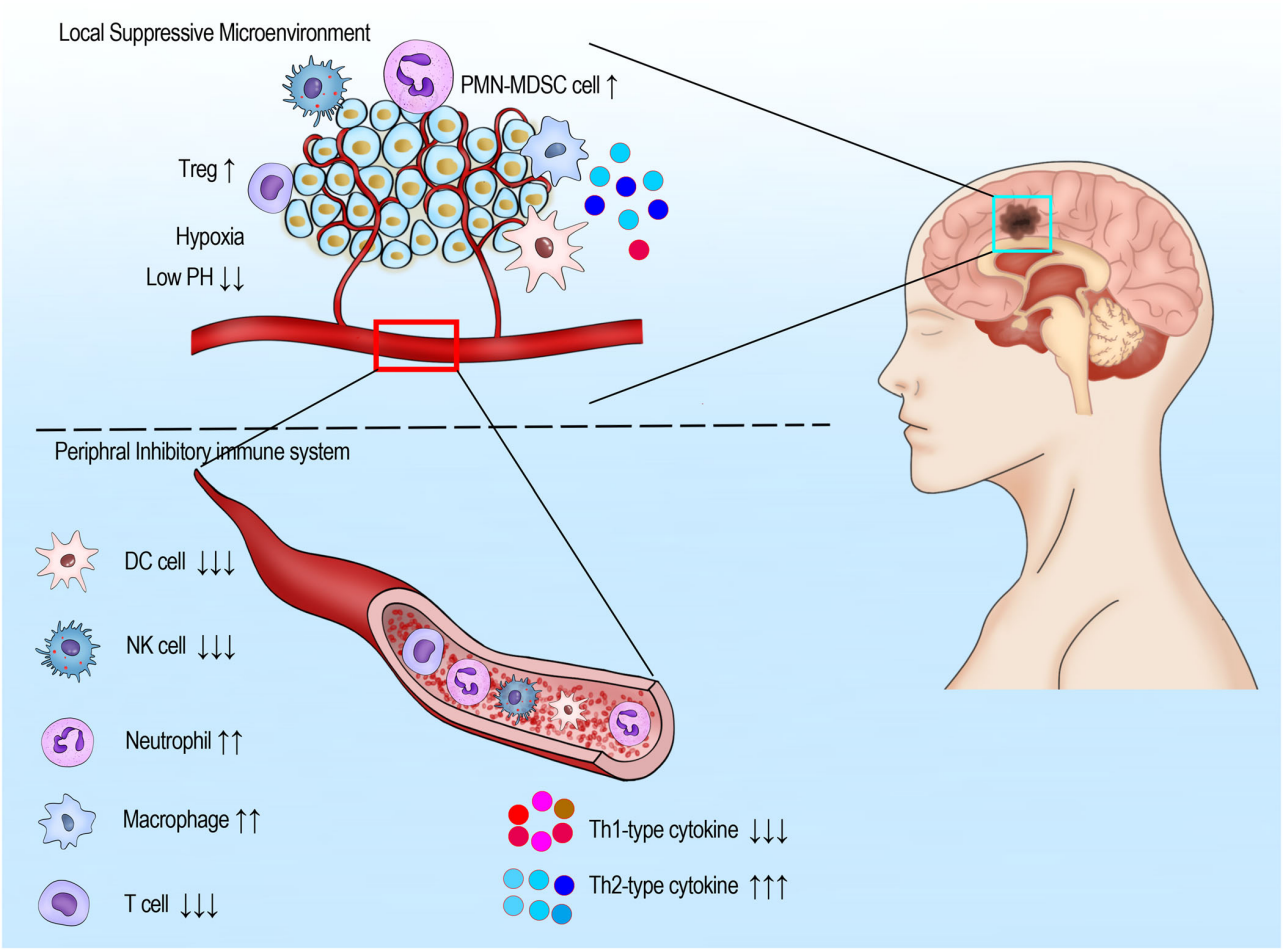
Systemic and local immunosuppression in glioma. Upper Panel: Local Immunosuppression. This panel focuses on the local tumor microenvironment of a glioma, highlighting the increased local recruitment and activation of immune suppressive cells such as MDSCs and macrophages. Moreover, there is a marked decrease in the activity of critical immune effector cells, including T cells and NK cells, at the tumor site, driven by the immunosuppressive signals within the microenvironment. These local dynamics contribute strongly to the ability of the tumor to evade immune surveillance and sustain its growth. Bottom Panel: Systemic Immunosuppression. This panel shows the overall decrease in systemic immune responsiveness in the context of glioma. Key features include a reduction in Th1‐type cytokines, which are crucial for mediating immunity against tumors. By contrast, Th2‐type cytokines, which generally promote humoral immunity, are elevated, contributing to an immunosuppressive milieu. This imbalance affects various immune cells, including T lymphocytes and NK cells, depicted by their decreased numbers. Additionally, the panel shows an increase in immunosuppressive cells, such as neutrophils and MDSCs, as well as macrophages, which further contribute to systemic immune suppression. DC, dendritic cell; MDSCs, myeloid‐derived suppressor cells; NK, natural killer; PMN, polymorphonuclear leukocytes; Th, T helper cell; Treg, regulatory T cell.

The brain TME comprises a heterogeneous and dynamic network composed of interacting immune, vascular, and resident cellular components [17]. Understanding the specific roles and interactions of these cellular components is crucial for developing novel therapeutic strategies to disrupt suppressive mechanisms within the TME. This disruption can potentially reactivate the ability of the immune system to combat GBM, thereby improving patient outcomes. The TME in GBM is highly heterogeneous. Different regions of the tumor can have varying levels of immune cell infiltration, cytokine profiles, and stromal components. This diversity presents a challenge for engineered immune cells to uniformly target and eliminate tumor cells. For example, tumor‐associated macrophages (TAMs) make up the majority of stromal cells in both low‐ and high‐grade GBM [18, 19]. Moreover, the GBM microenvironment is characterized by an upregulation of immune checkpoint molecules, such as programmed cell death ligand 1 (PD‐L1). PD‐L1 interacts with programmed cell death protein 1 (PD‐1) on T cells, leading to T‐cell exhaustion and reduced immune surveillance [20]. This complex system of immune evasion and suppression allows GBM cells to proliferate unchecked, evade immune destruction, and drive the aggressive progression of the disease. These alterations can provide valuable insights into both the response of the immune system to GBM and how this contributes to the overall disease progression. By understanding these changes, researchers can potentially develop more effective therapies to target and manipulate the immune system to combat GBM (Figure 1).

### 2.1 T Lymphocytes

T lymphocytes can serve as both positive and negative regulators of GBM growth, particularly in low‐grade GBM [21]. These cells engage in dynamic interactions with GBM cells and nontumor stromal cells, potentially either promoting or inhibiting tumor growth [22]. Patients with GBM often have deficiencies in both humoral and cellular immunity, with higher World Health Organization tumor grades correlating with greater immune impairment. Specifically, a decrease in the circulating cluster of differentiation (CD)3^+^ and CD19^+^ lymphocytes, a reduced CD4/CD8 ratio, and diminished levels of immunoglobulins and complement have been observed [23]. T‐cell lymphopenia in patients with GBM is predominantly attributed to the selective depletion of CD4^+^ cells. These abnormalities in T lymphocytes may be associated with the immune escape mechanisms used by GBM, allowing tumor cells to evade immune system attacks [24].

### 2.2 Neutrophils

Neutrophils are the most abundant type of circulating white blood cells and are primarily tasked with combating bacterial and fungal infections. However, they also play pivotal roles in the context of cancer [25]. Depending on their stage of maturation and activation, neutrophils can show both anti‐ and pro‐tumorigenic activities [26]. Their potential as clinical biomarkers and therapeutic targets is increasingly being recognized within the scientific community [27, 28]. The correlation between the neutrophil‐to‐lymphocyte ratio (NLR) and the prognosis of GBM and brain metastases has garnered substantial attention, and elevated NLR values have been established as predictors of poor overall survival. Patients with high NLR values frequently have high‐grade GBM, highlighting the efficacy of NLR as a prognostic indicator for tumor grading in patients with GBM [29]. Notably, only patients with increased peripheral blood neutrophil counts before bevacizumab treatment seem to derive benefit from this therapeutic intervention [30]. Targeting neutrophils may thus represent a novel approach in the next generation of immunotherapies, potentially enhancing the efficacy of cancer treatments [31].

### 2.3 Myeloid‐Derived Suppressor Cells (MDSCs)

MDSCs proliferate extensively during cancer, inflammation, and infection, showing a strong capacity to inhibit T‐cell responses [32]. Although the effects of MDSCs have been extensively studied in the context of GBM, relatively few investigations have focused on their role in peripheral blood. MDSC accumulation in the peripheral blood of patients with GBM may contribute to the suppression of T‐cell immunity [33]. Furthermore, a marked increase in the frequency of M2 macrophages and PD‐1^+^CD4^+^ T cells has been observed in the blood of patients with GBM [34]. Elevated plasma levels of arginase and granulocyte colony‐stimulating factor in patients with GBM may be associated with MDSC inhibition and amplification, respectively [33, 35]. Innovative therapeutic strategies, such as vascular endothelial growth factor‐A small interfering RNA‐doxorubicin‐loaded extracellular vesicle (EV)‐derived dendritic cells (DCs), have demonstrated promising results for inhibiting GBM growth, invasion, migration, and angiogenesis, while also eliciting effective anti‐tumor immune responses [36].

### 2.4 Macrophages

TAMs in GBM play a pivotal role in tumor progression by serving diverse metabolic and immunosuppressive functions within the TME [37, 38]. Their characteristics are shaped by metabolic signals in the TME, which enable them to interact closely with tumor cells, not only enforcing immunosuppression but also facilitating metabolic exchanges [39]. TAMs contribute to recycling myelin fragments, especially cholesterol‐rich components, which they convert into lipids that GBM cells use for energy and growth. GBM cells further exploit this interaction to enhance demyelination and remyelination processes by manipulating macrophages, leading to lipid accumulation in TAMs [40]. This metabolic support is particularly vital for mesenchymal‐like GBM cells, which often reside in hypoxic regions of the tumor and rely on external lipid sources for their survival and proliferation. These metabolic adaptations contribute strongly to tumor resilience, especially in recurrent cases. Lipid‐loaded macrophages, which are a subset of TAMs, play a key role in this process by showing strong immunosuppressive characteristics; these correlate with reduced efficacy of immune checkpoint inhibitors [41]. Together, these observations suggest that lipid‐loaded macrophages are central to tumor immune evasion and are critical drivers of GBM recurrence after treatment.

TAMs in brain cancers constitute a diverse assembly of immune cells, with notable variations in composition between primary and metastatic brain tumors [42]. Single‐cell analyses have revealed that primary brain tumors, and particularly GBM, are more commonly infiltrated by reactive microglia, which are distinguished by markers such as CD49d^–^MER proto‐oncogene, tyrosine kinase^+^CX3C motif chemokine receptor 1 (CX3CR1)^+^CD11c^+^CD64^+^. These microglia are dispersed throughout tumor regions but are conspicuously absent from the core of metastatic brain tumors originating from distant sites such as the breast or lung. Conversely, macrophages in both GBM and brain metastases tend to concentrate near CD31^+^ vascular structures [43]. These analyses further underscore the diversity of TAMs within GBM, revealing multiple subsets with unique gene signatures. For example, one subset comprises macrophages in a transitional state, marked by high expression of *LYZ*, *EREG*, and *S100A6* but low expression of *C1Q*. Another subset resembles microglia‐like macrophages, characterized by high expression of *BIN1*, *CX3CR1*, *TMEM119*, and *OLFML3*. Hypoxic macrophages are distinguished by high expression of *BNIP3*, *ADAM8*, *FAM162A*, and *MIF*, whereas phagocytic/lipid macrophages show high expression of *FABP5*, *GPNMB*, *LGALS3*, and *CD63*. The heterogeneity of TAMs in GBM is increasingly being recognized as context‐dependent and is influenced by various factors such as the origin of the tumor. Notably, microglia predominate in newly diagnosed GBM, whereas macrophages become more prevalent in recurrent GBM tumors [43]. This understanding of TAM heterogeneity in GBM has important implications for the development of targeted therapies and immune‐based treatments [44].

### 2.5 Microglia

Glioma‐associated microglia/macrophages (GAMs) encompass periphery‐derived macrophages and brain‐resident microglia, both of which contribute to glioma progression. The inflammatory mediator chemokine C─C ligand 5 (CCL5) has been implicated in tumor growth and migration across various cancer types, including glioma [45, 46]. However, the specific mechanisms through which CCL5 promotes glioma invasion remain largely unknown. To investigate this, researchers have used various experimental techniques such as wound healing assays, transwell assays, and three‐dimensional µ‐slide chemotaxis assays to assess glioma migration and invasion [47]. They have also used cytokine arrays, quantitative polymerase chain reaction, western blot analysis, and immunohistochemistry to measure the expression levels of CCL5, CD68, matrix metalloproteinase 2 (MMP2), phosphorylated (p‐)Ca^2+^/calmodulin‐dependent protein kinase II (CaMKII), p‐protein kinase B (Akt), and other relevant proteins. Additionally, zymography and intracellular calcium assays have been used to analyze MMP2 activity and intracellular calcium levels, respectively [35, 42].

The findings from these studies suggest that CCL5 modulates the migratory and invasive capabilities of human glioma cells in conjunction with MMP2 expression. In response to CCL5, glioma cells show a synchronized increase in intracellular calcium levels and p‐CaMKII and p‐Akt expression. Furthermore, the CCL5‐induced invasion of glioma cells and the corresponding increase in MMP2 are suppressed when p‐CaMKII is inhibited. Glioma cells tend to migrate toward GAM‐conditioned media activated by granulocyte–macrophage colony‐stimulating factor (GM‐CSF); this medium is abundant in CCL5. This homing effect is associated with MMP2 upregulation and can be mitigated by modulating intracellular and extracellular calcium levels or by antagonizing CCL5. Similarly, clinical data have revealed correlations between CCL5 levels and GAM activation. Overall, these findings suggest that the modulation of glioma CaMKII is a potential therapeutic target for reducing glioma growth by limiting the effects of CCL5 on glioma invasion. This concept presents a novel approach for the treatment of gliomas and other cancers in which CCL5 and related signaling pathways play a role in tumor progression [48].

### 2.6 Cytokines in Peripheral Blood and Cerebrospinal Fluid

Cytokines play a crucial role in the TME and can serve as prognostic indicators in patients with GBM [49]. T helper (Th)1 cytokines, such as tumor necrosis factor‐alpha (TNF‐α) and interferon (IFN)‐γ, are markedly lower in patients with GBM than in controls. By contrast, Th2 cytokines, including interleukin (IL)‐4 and IL‐10, are strongly expressed in both peripheral lymphocytes and GBM cell cultures [50]. Additionally, IL‐6 and IL‐8 have been associated with poorer overall survival in patients with GBM, whereas below‐median levels of transforming growth factor‐beta (TGF‐β) have been linked to increased survival. Together, these cytokines contribute to the establishment of a carcinogenic microenvironment that supports cancer progression and metastatic spread [51, 52].

The detection of cytokines in cerebrospinal fluid is also crucial for the diagnosis and prognosis of GBM. Various cytokines in the cerebrospinal fluid, such as TNF‐α, TGF‐β, IFN, IL‐2, IL‐4, IL‐6, IL‐8, IL‐10, IL‐12, and IL‐13, play roles in shaping the GBM microenvironment [53]. Additionally, the cerebrospinal fluid concentration of IL‐6 is correlated with the TAM invasion level and may serve as a useful prognostic biomarker in patients with GBM [54].

## 3 Importance of Cellular Immune Diagnosis and Treatment

Targeting the TME in GBM presents important challenges because of the intricate barriers that impede immune cell infiltration and function [21, 55]. These include the dense extracellular matrix, physical pressure within the tumor, immunosuppressive cytokines and chemokines, and the recruitment and expansion of immunosuppressive cells [56, 57]. The effects of GBM on patients are multifaceted and profound. As the tumor enlarges, it can lead to increased intracranial pressure, potentially causing brain displacement. In severe cases, this may result in brain herniation, posing a major threat to the patient's life. Furthermore, GBM can cause a variety of focal symptoms depending on its location, including memory loss, mood alterations, psychiatric symptoms, language disorders, and personality changes. Additionally, neurological symptoms such as seizures and psychiatric disorders may also manifest [58].

**Figure 2:**
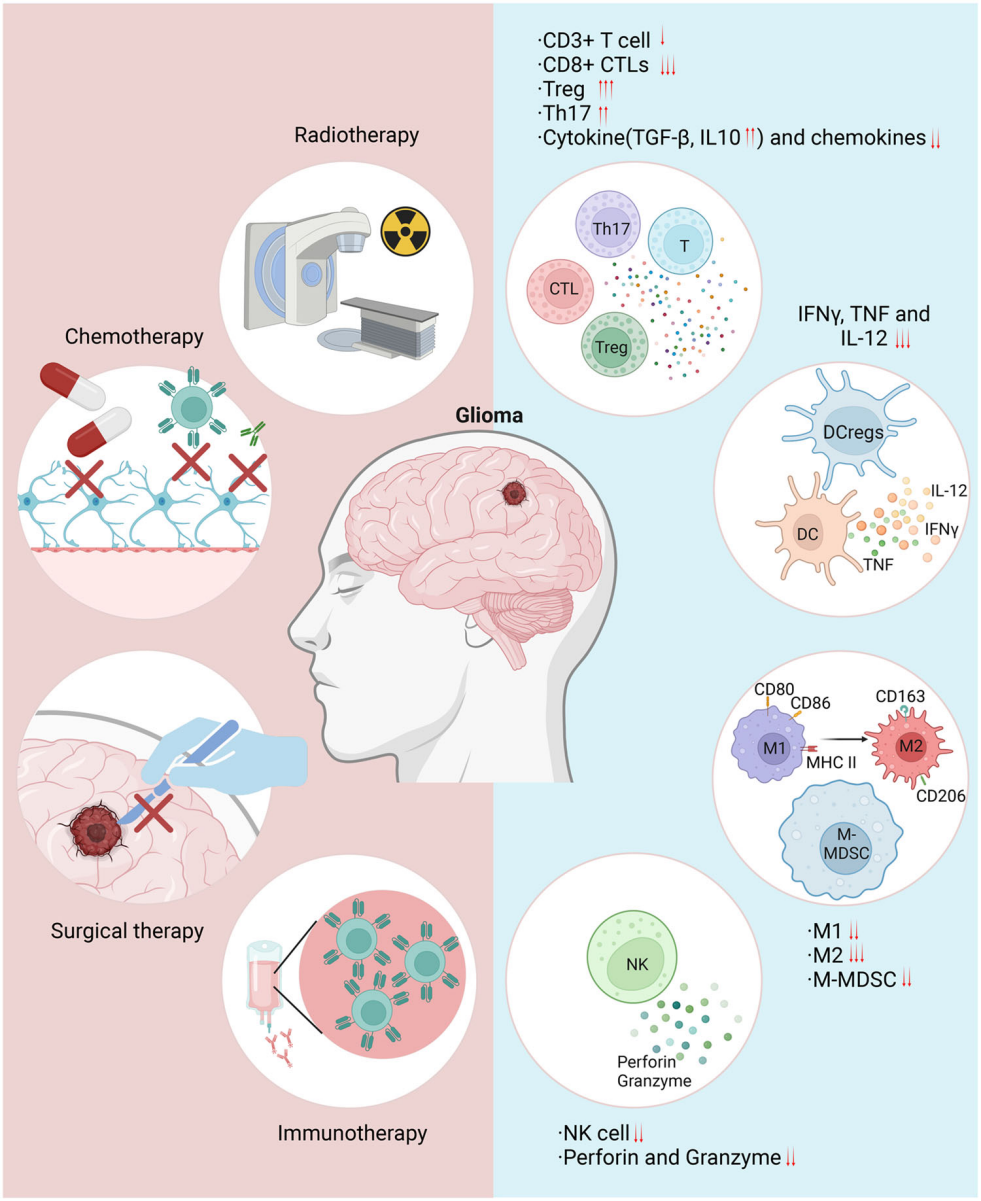
Multimodal treatment strategies in glioma. Surgical intervention: This intervention constitutes the initial approach to glioma treatment, using surgical techniques such as resection or debulking to reduce tumor mass. This panel includes imagery of neurosurgical procedures, tools used in the operation, and the mapping of brain regions to avoid critical areas during surgery. Radiation therapy: Radiation therapy is often applied post‐surgery; targeted radiation is used to eliminate residual tumor cells that are inaccessible surgically. This panel displays types of radiation techniques, such as external beam radiation therapy, stereotactic radiosurgery, or proton therapy, detailing their precision and impact on the tumor and surrounding tissues. Chemotherapy and targeted drug therapy: Chemotherapeutic and targeted drug treatments are often used in glioma management. This panel details common chemotherapeutic agents, such as temozolomide, and targeted therapies such as bevacizumab (anti‐vascular endothelial growth factor). The panel also shows the mechanisms of action of these drugs at the cellular level and their systemic administration. Immunotherapy and emerging treatments: Advanced treatment options include immunotherapy, such as checkpoint inhibitors, vaccines, or adoptive cell therapies, such as CAR‐T cell therapy. Emerging treatments such as tumor‐treating fields are also depicted, demonstrating the novel approaches being explored to enhance treatment efficacy and patient outcomes. CAR‐T, chimeric antigen receptor T cells; CD, cluster of differentiation; CTLs, cytotoxic T lymphocytes; DC, dendritic cell; DCregs, regulatory dendritic cells; IFN‐γ, interferon‐gamma; IL, interleukin; MHC, major histocompatibility complex; M‐MDSCs, monocytic myeloid‐derived suppressor cells; NK, natural killer; TGF‐β, transforming growth factor‐beta; Th, T helper cell; TNF, tumor necrosis factor; Treg, regulatory T cell.

The treatment of GBM necessitates a multidisciplinary approach, incorporating surgery, radiation therapy, chemotherapy, and immunotherapy (Figure 2). Surgical resection is typically the preferred initial treatment, aiming to remove as much of the tumor as possible [59, 60]. However, because of the infiltrative nature and indistinct borders of GBM, complete resection is often challenging, leading to residual disease post‐surgery. Given the high recurrence rate of GBM, chemotherapy and radiation therapy are frequently used to inhibit tumor growth, alleviate symptoms, and reduce the risk of recurrence. However, the effectiveness of chemotherapy is often limited because chemotherapeutic agents often have difficulties crossing the BBB, thus diminishing their effects on GBM [60].

Recently, immunotherapy has emerged as a promising treatment option for GBM [61]. By stimulating a patient's immune system to target tumor cells, immunotherapy can inhibit tumor growth and improve clinical symptoms [5]. However, the current scope of targeted therapies for GBM remains limited, which constrains their overall therapeutic efficacy. Consequently, the development of novel and more effective targeted therapeutic agents is a critical focus for advancing GBM treatment [62].

## 4 Emerging Cellular Immune Diagnostic Approaches

Emerging cellular immune diagnostic techniques are revolutionizing GBM diagnosis by providing more precise and personalized insights into tumor biology [63]. These techniques focus on analyzing the immune environment within and around the tumor, thereby offering potential biomarkers that can aid in diagnosis, prognosis, and treatment planning [64, 65]. One such technique is flow cytometry, which allows for the detailed characterization of immune cell populations in tumor samples and peripheral blood. It offers valuable information about the immune landscape and potential immunotherapy targets. Another promising approach is single‐cell RNA sequencing, which enables the profiling of gene expression at the single‐cell level, revealing the heterogeneity of immune cells within the TME and identifying novel immune markers [66]. Additionally, multiplex immunohistochemical and immunofluorescent techniques are increasingly being used to simultaneously detect multiple immune markers in tissue sections, providing a more comprehensive understanding of the spatial distributions and interactions of immune cells within tumors [67, 68].

Although these techniques offer strong advantages in terms of specificity and the ability to identify new biomarkers, they also present challenges such as high costs and a need for specialized equipment and expertise, and are limited in their scalability for routine clinical use. A comparison of cellular immune diagnostic approaches is summarized in Table 1. These emerging techniques are pushing the boundaries of GBM diagnosis, offering detailed and actionable information; however, careful consideration of their limitations is required before their widespread adoption in clinical practice.

**Table 1:** Cellular immune diagnostic approaches.

Technique	Advantages	Disadvantages	References
Flow cytometry	Rapid analysis, detailed characterization of immune cell populations.	Requires fresh samples, provides limited spatial information, and uses costly reagents.	[69, 70, 71]
Single‐cell RNA sequencing	High resolution, identifies cellular heterogeneity and novel biomarkers.	Expensive, needs complex data analysis, and requires specialized expertise.	[66, 72]
Multiplex immunohistochemistry/immunofluorescence	Simultaneous detection of multiple markers, provides spatial context.	Technically demanding, has potential for cross‐reactivity, and high cost.	[67, 73]

There are several challenges and limitations to the diagnosis of GBM; these can hinder its precise identification and classification [68]. A key challenge lies in the intrinsic heterogeneity of GBM, meaning that different areas within the same tumor can display diverse cellular and molecular features. This heterogeneity complicates the process of obtaining a biopsy sample that accurately represents the entire tumor. Moreover, although advanced imaging methods such as magnetic resonance imaging (MRI) and positron emission tomography (PET) provide valuable information about tumor shape and metabolic activity, they cannot always differentiate between tumor tissue and surrounding inflammatory responses or treatment‐induced changes. This distinction is crucial because misinterpretation can lead to incorrect diagnoses [74]. Another important limitation is the reliance on histopathological examination, which—although considered the gold standard for diagnosis—can be interpreted differently by different observers. Furthermore, histopathological analysis may not fully capture the complex molecular characteristics of GBM. The ongoing development of GBM subtype classification and the integration of molecular diagnostics, therefore, offer promising avenues for improved understanding and diagnosis. However, these approaches face practical hurdles, including access to testing, technique standardization, and accurate interpretation of results. These challenges are particularly pronounced in cases of low‐grade GBM, in which slow growth and subtle imaging features can further delay diagnosis. Collectively, these various factors emphasize the need for ongoing advances in diagnostic tools and methodologies to enhance the accuracy, consistency, and timeliness of GBM diagnosis.

## 5 Cellular Immune Therapeutic Approaches for GBM

In GBM treatment, breakthroughs achieved by immunotherapy have garnered attention from the medical community and patient groups alike [4]. Recent research and clinical trial data indicate that immunotherapy can effectively inhibit tumor growth and spread, ultimately improving patient survival rates and quality of life [16]. However, despite the potential of immunotherapy in GBM treatment, several challenges remain, including immune escape, drug resistance, and the sustainability of treatment effects [75]. Researchers and clinicians must therefore conduct more in‐depth studies and clinical trials to enhance the application of immunotherapy in GBM treatment, thereby providing more therapeutic options and better outcomes for patients [76]. As a novel treatment approach, immunotherapy has increasingly drawn attention in the context of GBM treatment. The following sections highlight some recent advances in immunotherapy for GBM.

### 5.1 Immune Checkpoint Inhibitors

One key advance in immunotherapy for GBM is the use of immune checkpoint inhibitors. These inhibitors target specific proteins on immune and tumor cells that play a role in immune suppression. For example, PD‐L1 is often upregulated on tumor cells to evade the immune response. By blocking PD‐L1 with monoclonal antibodies, researchers can disrupt its interaction with PD‐1 on cytotoxic T lymphocytes, allowing these cells to effectively recognize and destroy tumor cells [77]. Similarly, blocking cytotoxic T lymphocyte‐associated protein 4 (CTLA‐4), an inhibitory immune checkpoint molecule that binds to CD80 and CD86 and prevents their interaction with CD28, can enhance T‐cell priming by DCs.

Despite the promising potential of immune checkpoint inhibitors in GBM treatment, several challenges remain. The expression of PD‐L1 in GBM is variable, and its importance as a biomarker for sensitivity to anti‐PD‐1 therapy is controversial [78]. Additionally, the unique immune landscape of the brain contributes to the complexity of the tumor immune microenvironment in GBM.

Recent research has begun to unravel the complexity of the GBM tumor immune microenvironment. Single‐cell sequencing studies have revealed that microglia (the resident immune cells of the brain) undergo severe oxidative stress, leading to the activation of nuclear receptor subfamily 4 group A member 2 (NR4A2)‐dependent transcriptional activity. By targeting NR4A2 or its downstream effector, squalene monooxygenase, researchers can alter microglial plasticity and improve the efficacy of immune checkpoint blockade in vivo [79]. Another study by Liu et al. [80] analyzed the TME in gliomas and reported that tumor‐infiltrating T cells are primarily restricted to perivascular cuffs and express high levels of C─C motif chemokine receptor 5, C─X─C chemokine receptor (CXCR)3, and PD‐1. These T cells are predominantly classified into pre‐exhausted/exhausted and effector CD8^+^ T cell subsets, along with cytotoxic CD4^+^ T cell subsets. Notably, a distinct subpopulation of CD4^+^ T cells shows innate‐like characteristics and favor IL‐8 expression. Blocking IL‐8 with antibodies or inhibiting its receptor, CXCR1/2, enhances the anti‐tumor response mediated by anti‐PD‐1 therapy. Together, these findings suggest that IL‐8 represents a promising target for combination immunotherapy in glioma.

Although immunotherapy has shown considerable promise in the treatment of GBM, there are still many challenges to overcome. In‐depth studies and clinical trials that aim to enhance the application of immunotherapy in GBM treatment should be further investigated to provide more therapeutic options and better outcomes for patients [80].

### 5.2 Personalized Tumor Vaccines

Tumor‐specific antigens are crucial for stimulating the immune system to attack tumors; this principle is being explored in personalized vaccine therapies for GBM. Various strategies are used in cancer vaccination. One approach involves transferring a tumor‐specific antigen (or a combination of these) to antigen‐presenting cells, which then present to T cells to elicit an immune response [81, 82]. However, this method is limited by its specificity to human leukocyte antigen subtypes and depends on the expression of specific antigens in the tumor.

Rindopepimut, a vaccine targeting epidermal growth factor receptor variant III (EGFRvIII), is the only vaccine treatment that has been subjected to comprehensive evaluation [83]. Unfortunately, it failed to improve survival when used in combination with standard therapy for newly diagnosed EGFRvIII‐mutant patients with GBM. Other vaccines have shown promising results in trials; for example, heat‐shock protein peptide complex‐96 was reported to have a 6‐month survival rate of 90% in a phase II trial, and autologous formalin‐fixed tumor vaccine had a 3‐year survival rate of 38% in a phase I/IIa trial. GBM vaccine therapy primarily relies on DCs to present GBM‐associated peptides, antigens, or tumor epitopes to T cells of the adaptive immune system. The basic principle involves isolating specific antigens from tumor cells and processing them into human leukocyte antigen‐matched vaccines. However, although the EGFRvIII‐targeted peptide vaccine Rindopepimut showed potential in phase II trials, it did not deliver the desired survival benefits in phase III trials. By contrast, DC vaccines appear more promising because of their mechanisms of T‐cell activation. DC vaccines prepared from the pulsed lysate of each patient's own glioma cells have been reported to significantly improve the overall survival of glioma patients in a phase III trial [84, 85]. Furthermore, the multi‐target compound vaccine IMA‐950 extended the median survival of patients in a phase I trial. Bvax, a B‐cell‐based vaccine, has also shown therapeutic responses in preclinical models of GBM by inhibiting key processes such as GBM cell migration and invasion [86, 87].

Currently, the preparation of personalized neoantigen vaccines is a leading trend in vaccine immunotherapy. Pre‐vaccination with bacterial‐derived outer membrane vesicles can enhance the anti‐tumor effects of vaccine treatment in other contexts; however, no clinical trials have been conducted as yet for glioma treatment [88]. Overall, the field of GBM vaccine therapy is evolving, with various promising candidates and strategies under investigation.

### 5.3 CAR‐T Cell Therapy

CAR‐T cells are a form of immunotherapy known for their strong ability to target and kill specific cancer cells [89]. CAR‐T cell therapy has been explored as a potential treatment for GBM; it is particularly advantageous because it bypasses the need for major histocompatibility complex (MHC)‐dependent co‐stimulation by fusing the antigen‐binding domain with the T‐cell activation and co‐stimulatory domains [90, 91]. This approach has shown impressive clinical responses, particularly in patients with refractory lymphoma treated with CD19‐targeted CAR‐T cells, leading to the first approved CAR therapy [92]. However, the potent immune response generated by CAR‐T cells can result in severe side effects, such as cytokine release syndrome (CRS), which has led to fatalities. In solid tumors, off‐target toxicity can cause severe organ dysfunction, limiting the use of highly tumor‐specific antigens [93]. Moreover, the TME in solid tumors such as GBM diminishes CAR‐T cell infiltration, proliferation, and persistence.

Intratumoral myeloid cells—particularly, blood‐borne myeloids (BBMs)—play a crucial role in driving T‐cell dysfunction within the TME. MHC class II (MHC‐II)‐restricted antigen presentation on the BBM is essential for controlling brain tumor growth. The absence of MHC‐II on BBM leads to dysfunctional T cells, characterized by increased chromatin accessibility and the upregulation of thymocyte selection‐associated high mobility group box protein TOX (a key regulator of T‐cell exhaustion). This MHC‐II‐restricted antigen presentation is essential for maintaining the functional states of cytotoxic T cells in brain tumors. Mechanistically, CD4^+^ T cell activation through MHC‐II‐dependent pathways suppresses myeloid‐derived osteopontin, which otherwise induces the activation of the chronic nuclear factor of activated T cells, cytoplasmic 1, in tumor‐reactive CD8^+^ T cells. This MHC‐II‐restricted antigen presentation on the BBM is fundamental for maintaining the functional states of cytotoxic T cells in brain tumors [94]. In an academic phase I–II clinical trial, GD2‐CAR‐T cells, known as GD2‐CART01, were evaluated in children with relapsed or refractory high‐risk neuroblastoma. The trial demonstrated that GD2‐CART01 therapy is feasible and safe, with promising efficacy. GD2‐CAR‐T cells showed robust in vivo expansion and prolonged persistence in the majority of patients, with 63% of patients responding to therapy. This trial underscores the potential of CAR‐T cell therapy for high‐risk neuroblastoma, particularly with the use of an inducible suicide gene to mitigate severe side effects [95].

Building on previous findings that the disialoganglioside GD2 is highly expressed on H3K27M‐mutated glioma cells, a first‐in‐human phase I clinical trial (NCT04196413) was initiated to explore GD2‐CAR‐T cell therapy in patients with H3K27M‐mutated diffuse intrinsic pontine glioma or spinal cord diffuse midline glioma. Early clinical results were promising, highlighting the potential of GD2‐CAR‐T cell therapy for treating these historically treatment‐resistant diseases [96]. In a mouse syngeneic GBM model, CAR‐T cell therapy not only activated intratumoral myeloid cells but also induced endogenous T‐cell memory responses, amplifying CAR‐T cell activity. The production of IFN‐γ by CAR‐T cells and the responsiveness of host immune cells to IFN‐γ were essential for reshaping the tumor immune landscape, leading to more activated and less suppressive TME. These findings suggest that CAR‐T cell therapy can transform the TME, supporting the activation of endogenous anti‐tumor immunity and highlighting the critical role of IFN‐γ signaling in the success of CAR‐T cell therapy for GBM [97].

Meister et al. [98] developed multifunctional CAR‐T cells that co‐express a multitargeting CAR derived from the NK group 2D (NKG2D) receptor alongside pro‐inflammatory cytokines IL‐12 and IFN‐α2. These cells demonstrate promising anti‐glioma activity in vitro and in mouse models without any signs of toxicity. Additionally, an oncolytic adenovirus armed with the chemokine CXCL11 has been used to enhance CAR‐T cell infiltration and reprogram the immunosuppressive TME in GBM. Wang et al. [99] developed an oncolytic adenovirus armed with the chemokine CXCL11 to enhance CAR‐T cell infiltration and reprogram the immunosuppressive TME in GBM. The combination of B7 homolog 3 (B7H3)‐targeted CAR‐T cells and CXCL11‐armed oncolytic adenovirus achieved a sustained anti‐tumor response and reprogrammed the TME, increasing the infiltration of CD8^+^ T cells, NK cells, and M1‐polarized macrophages while reducing immunosuppressive cell populations. This approach shows promise as a powerful adjuvant to CAR‐T therapy for GBM. Weiss et al. [100] investigated NKG2D‐based CAR‐T cells in GBM mouse models and demonstrated robust IFN‐γ production, strong cytolytic activity, and the ability to migrate to the brain tumor site, extending survival and even curing some glioma‐bearing mice. Notably, the combination of NKG2D‐based CAR‐T cells with subtherapeutic doses of radiotherapy enhanced CAR‐T cell migration and effector functions, providing a strong rationale for translating this approach into human patients with glioma [101].

### 5.4 CAR‐NK Therapy

CAR‐NK therapy is an innovative form of immunotherapy that leverages the natural ability of NK cells to target and kill cancer cells [102]. By genetically engineering NK cells to express CARs, which are designed to recognize specific antigens on the surface of cancer cells, CAR‐NK therapy augments the cytotoxic activity of NK cells against tumors. Unlike CAR‐T cells, CAR‐NK cells have several advantages, including a diminished risk of severe side effects (such as CRS and graft‐vs.‐host disease) and their potential as an “off‐the‐shelf” therapy because of the reduced likelihood of immune rejection [102, 103]. CAR‐NK therapy is currently being investigated for treating various cancers, including GBM, with promising outcomes in preclinical and early clinical trials.

Strecker et al. [104] evaluated the effectiveness of the tumor‐specific delivery of an anti‐PD‐1 immunoadhesin (aPD‐1) using a targeted adeno‐associated viral vector (AAV) in conjunction with receptor protein‐tyrosine kinase (HER2)‐specific NK‐92/5.28.z (anti‐HER2.CAR/NK‐92) cells as a component of a combination immunotherapy strategy. In co‐culture experiments, target‐activated anti‐HER2.CAR/NK‐92 cells modified both adjacent tumor cells and bystander immune cells by inducing the secretion of inflammatory cytokines and upregulating PD‐L1. The specific delivery of aPD‐1 to tumor cells was facilitated by displaying a HER2‐specific designed ankyrin repeat protein on the AAV surface. The efficiency of HER2‐AAV‐mediated gene transfer into GBM cells was correlated with HER2 expression levels without eliciting antiviral responses in the transduced cells. Furthermore, AAV transduction did not interfere with tumor cell lysis mediated by anti‐HER2.CAR/NK‐92 cells. Following the selective transduction of HER2^+^ cells, aPD‐1 expression was confirmed at both mRNA and protein levels. This approach offers a promising strategy for GBM immunotherapy by potentially enhancing efficacy while mitigating the systemic side effects associated with immune checkpoint inhibitors.

Kong et al. [105] explored targeting the GBM TME—specifically, pericytes expressing CD19—to evaluate the effectiveness of CD19 CAR‐induced pluripotent stem cell‐derived NK (iNK) cells against GBM. Using GBM–blood vessel assembloids, which combine GBM spheroids with blood vessel organoids, iNK cells engineered to express CD19 CAR demonstrated targeted migration toward pericytes surrounding the GBM. A microfluidic chip confirmed the targeted action and cytotoxicity of CD19 CAR‐iNK cells in a perfusion‐like environment. In vivo, GBM–blood vessel assembloid xenografts mimicking the TME, including human CD19^+^ pericytes, confirmed the efficacy of CD19 CAR‐iNK cells against GBM. The presence of pericytes significantly increased CD19 CAR‐iNK cell migration toward GBM and reduced tumor cell proliferation, thus highlighting the therapeutic potential of CD19 CAR‐iNK cells for targeting pericytes within the GBM TME.

Chaudhry et al. [106] developed an off‐the‐shelf CAR‐NK cell approach utilizing a B7H3 CAR, and demonstrated that CAR‐transduced NK cells show strong cytolytic activity against GBM cells in vitro. However, the presence of TGF‐β in the TME significantly impairs the cytolytic function of both unmodified and CAR‐transduced NK cells. To counteract this immune suppression, NK cells were co‐transduced with both a B7H3 CAR and a TGF‐β dominant‐negative receptor, thus successfully preserving their cytolytic activity even in the presence of exogenous TGF‐β. These results suggest that the co‐expression of a dominant‐negative receptor and CAR represents a promising therapeutic strategy for treating CNS tumors such as GBM.

Burger et al. [107] conducted a phase I first‐in‐human clinical trial to assess the safety and feasibility of the adoptive transfer of clonal CAR‐NK cells (NK‐92/5.28.z) targeting HER2, which is overexpressed in a subset of GBM. The treatment was well tolerated, with no dose‐limiting toxicities and no observed instances of CRS or immune effector cell‐associated neurotoxicity syndrome (ICANS). Post‐treatment, five patients experienced stable disease for 7–37 weeks, whereas four patients had progressive disease. Notably, higher levels of CD8^+^ T‐cell infiltration in recurrent tumor tissue before CAR‐NK cell injection were positively correlated with longer time to progression. These findings indicate that the intracranial injection of HER2‐targeted CAR‐NK cells is feasible and safe in patients with recurrent GBM.

### 5.5 CAR‐NK T Cells (NKTs)

The current interim report presents the latest findings from a first‐in‐human phase 1 clinical trial assessing the safety and efficacy of autologous NKTs engineered to co‐express a GD2‐specific CAR and IL‐15, referred to herein as GD2‐CAR.15. Twelve pediatric patients diagnosed with neuroblastoma were enrolled in the trial. The primary objectives were to evaluate the safety profile and determine the maximum tolerated dose of the therapy. The secondary objectives included the assessment of the anti‐tumor activity of GD2‐CAR.15 NKTs and the monitoring of immune responses. Encouragingly, no dose‐limiting toxicities were observed, and the maximum tolerated dose was not reached, suggesting a favorable safety profile. Notably, only one patient experienced grade 2 CRS, which was effectively managed with tocilizumab. The therapy demonstrated a promising objective response rate of 25%, with two patients achieving partial responses and one achieving a complete response. Further analysis revealed that the frequency of CD62L^+^ NKTs in the administered products was positively correlated with CAR‐NKT expansion in patients, and this expansion was notably higher in responders (defined as patients with either an objective response or stable disease accompanied by a reduction in tumor burden). An important mechanistic insight emerged with the observation that BTG anti‐proliferation factor 1 (BTG1), a protein associated with cellular hyporesponsiveness in exhausted NKT and T cells, was upregulated in peripheral GD2‐CAR.15 NKTs. Importantly, knockdown of BTG1 in GD2‐CAR.15 NKTs led to the elimination of metastatic neuroblastoma in a preclinical mouse model, suggesting a potential strategy for enhancing the anti‐tumor efficacy of this therapy. These results underscore the safety and clinical potential of GD2‐CAR.15 NKTs in treating pediatric neuroblastoma and highlight the possibility of further augmenting their efficacy through targeting BTG1. Such strategies may offer more effective treatments for this challenging malignancy. The study is registered on ClinicalTrials.gov under the identifier NCT03294954 [108, 109].

### 5.6 Macrophage Therapy

Immunosuppressive M2‐phenotype macrophages can be reprogrammed into tumor‐killing M1‐phenotype macrophages, a strategy that has been a focal point in anti‐tumor therapy [110]. The colony‐stimulating factor 1(CSF‐1)/CSF‐1 receptor (CSF‐1R) pathway facilitates the proliferation of TAMs and promotes M2 macrophage polarization [111]. However, the CSF‐1R inhibitor PLX3397, although tolerable, did not demonstrate therapeutic efficacy in a phase II trial [112]. Subsequently, other researchers compared different CSF‐1R inhibitors, including GW2580, PLX3397, and BLZ945, and reported that GW2580 may have potential as a new clinical trial candidate [113]. Moreover, the use of an agonist of stimulator of IFN genes, zinc–cyclic dimeric adenosine monophosphate, can help to convert M2 macrophages into the M1 phenotype, potentially transforming cold tumors into hot tumors [114]. Additionally, downregulating miR‐142‐3p during M1 to M2 polarization can block TGF‐β1, thereby inhibiting glioma growth and prolonging survival [115].

GBM stem cells (GSCs) that persist following surgical resection have a marked capacity for self‐renewal, playing a pivotal role in glioma recurrence. CD133, a widely recognized marker of cancer stem cells, is closely associated with the expression of anti‐apoptotic proteins and is a key factor in glioma recurrence. In response to this challenge, researchers have developed a hydrogel superstructure containing a nanotransporter loaded with a CAR plasmid. This hydrogel is administered into the glioma surgical resection cavity, where it facilitates the In Situ genetic recombination of inhibitory macrophage genes into CD133‐CAR macrophages. These CAR‐modified macrophages are then able to specifically target and phagocytose GSCs, thereby mitigating the risk of glioma recurrence. Experimental results indicate that combining a nanotransporter loaded with a CAR plasmid with anti‐CD47 antibodies enhances the anti‐tumor response, yielding even stronger therapeutic effects. This approach circumvents the complexities and high costs typically associated with conventional CAR‐T cell therapies and introduces an innovative method for intracavitary delivery, which may improve patient compliance. To date, however, this strategy has only been tested in a humanized GBM mouse model, and further clinical trial data are necessary to validate its efficacy [116].

Tracking and eliminating residual postsurgical GSCs is critical for preventing GBM relapse; however, effective strategies remain elusive. In this context, a novel cavity‐injectable nanoporter–hydrogel superstructure, designed to generate GSC‐specific CAR macrophages/microglia within the surgical cavity, has been introduced. The hydrogel‐based nanoporter reportedly delivers CAR genes specifically targeting GSCs into the nuclei of macrophages following intracavitary administration in mouse models of GBM. The CAR macrophages/microglia then actively seek out and engulf GSCs, thereby clearing residual tumor cells and promoting an adaptive anti‐tumor immune response within the TME [116] (Table 2).

**Table 2:** CAR‐T/NK tumor‐targeting therapy for GBM.

Target	Description	Study/trial details	References
EGFRvIII	CAR‐T cells targeting EGFRvIII regulated by synNotch, with stronger anti‐tumor effects than traditional CAR‐T cells.	Phase I trial: CARv 3‐TEAM‐ET cells targeting EGFRvIII were injected into the cerebrospinal fluid of recurrent patients with GBM, showing significant tumor volume reduction.	[117, 118]
IL‐13Rα2	CAR‐T cells targeting IL‐13Rα2.	Phase I trial (NCT02208362): CAR‐T cells delivered via intratumoral and intraventricular administration; combined intraventricular and intratumoral administration showed best results, improving the median survival of patients with recurrent glioma.	[119]
EGFR and IL‐13Rα2	Bivalent tandem CAR‐T cells targeting both EGFR and IL‐13Rα2 for highly heterogeneous tumors.	Phase I trial (NCT05168423): injected into cerebrospinal fluid, showing strong cytotoxicity and reducing glioma volume; however, neurotoxicity was observed in all six patients.	[120]
NKG2DL	CAR‐T cells targeting the NKG2D ligand, which is overexpressed on GBM stem cells.	Preclinical studies: CAR‐T cells showed the ability to lyse glioma stem cells both in vitro and in vivo using a mouse model derived from glioma xenografts.	[101, 121]
B7H3	CAR‐T cells targeting B7H3, administered repeatedly to patients with diffuse intrinsic pontine glioma.	Phase I trial (NCT04185038): determined the optimal dose level (1 × 10^7^ cells), observed sustained expression of B7H3 CAR‐T cells in cerebrospinal fluid.	[122]
Disialylganglioside GD2	CAR‐T cells targeting GD2, used to treat relapsed/refractory neuroblastoma.	Phase I trials (NCT02761915, NCT02173093): demonstrated safety and anti‐tumor effects; sustained CAR‐T expression was observed in high‐dose groups, and there was effectiveness with GD2Bi‐armed activated autologous T cells, despite cytokine release syndrome in all patients.	[96, 123, 124, 125]
CLTX	CLTX binds to chloride channels and MMP2 of malignant glioma cells, inhibiting glioma cell occurrence without causing cytotoxicity.	Preclinical studies: CLTX CAR‐T cells showed broad ability to target GBM in a mouse model, addressing the issue of off‐target effects caused by the lack of tumor‐specific antigens in gliomas.	[126, 127]

Abbreviations: B7H3, B7 homolog 3; Bi, bispecific antibody; CAR‐T, chimeric antigen receptor T cells; CLTX, chlorotoxin; EGFR, epidermal growth factor receptor; EGFRvIII, epidermal growth factor receptor variant III; GBM, glioblastoma multiforme; IL‐13Rα2, interleukin‐13 receptor alpha 2; MMP2, matrix metalloproteinase 2; NK, natural killer; synNotch, synthetic Notch; and TEAM, T‐cell–engaging antibody molecule.

### 5.7 OVs

OVs are either naturally occurring or genetically engineered viruses that induce anti‐tumor responses by selectively replicating within cancer cells, leading to cell lysis or immunogenic cell death [128]. GBM has been demonstrated to be susceptible to OVs in preclinical models. To overcome the challenge posed by the BBB, OVs are often administered directly into the tumor or use viruses with strong CNS penetration, such as parvovirus [129]. Although early clinical trials have demonstrated the safety of OVs in treating GBM, their therapeutic efficacy remains modest to date.

#### 5.7.1 Adenovirus

DNX‐2401 (also known as Delta‐24‐RGD), a second‐generation oncolytic adenovirus, has received US Food and Drug Administration approval for its promising therapeutic potential. This virus is genetically engineered with a 24‐base pair deletion in the *E1A* gene and modifications to the RGD sequence within the fiber region. These alterations allow DNX‐2401 to block the retinoblastoma signaling pathway and selectively bind to integrins on tumor cells [130]. Consequently, DNX‐2401 exerts potent oncolytic effects and enhances immune cell infiltration around the tumor [131].

Notably, when DNX‐2401 is combined with the PD‐1 inhibitor pembrolizumab, it significantly prolongs the survival time of glioma patients [132]. This combination therapy highlights a promising new approach, providing innovative strategies for the treatment of GBM by leveraging the synergistic effects of oncolytic virotherapy and immune checkpoint inhibition.

#### 5.7.2 Herpes Simplex Virus (HSV)‐1

A notable strategy in oncolytic virotherapy involves using adenoviruses to induce apoptosis in tumor cells by expressing HSV thymidine kinase, which phosphorylates ganciclovir and disrupts DNA replication [133]. Phase I trials have demonstrated that the dual virus vectors Ad‐hCMV‐TK and Ad‐hCMV‐Flt3L are both safe and tolerable [134]. Furthermore, researchers have engineered an AAV vector targeting the brain endothelium; it alters glioma vasculature through the expression of LIGHT protein and enhances T cell infiltration into tumor cells, providing a novel perspective for glioma treatment [135].

In a phase II single‐arm trial [136], G47Δ (Teserpaturev), a third‐generation HSV‐1 OV modified from the G207 vector, demonstrated promising therapeutic potential. The genetic modifications in G47Δ include the deletion of two copies of the γ34.5 and α47 genes, along with the insertion of *lacZ* into the *ICP6* gene, which causes its inactivation [137]. MRI‐guided stereotactic injections of G47Δ into gliomas resulted in prolonged median overall survival. Repeated injections of G47Δ also led to increased tumor‐infiltrating T cells, suggesting an enhanced immune response against the tumor [138]. Ling et al. [128] reported findings from a first‐in‐human phase I trial (NCT03152318) involving 41 patients with recurrent GBM who received injections of CAN‐3110, an oncolytic HSV. Unlike other clinical oncolytic HSVs, CAN‐3110 retains the viral neurovirulence ICP34.5 gene, which is transcribed by a nestin promoter. Nestin is significantly overexpressed in GBM and other invasive tumors but not in adult brain tissue or healthy differentiated cells. This genetic modification allows CAN‐3110 to replicate within tumor cells preferentially. The trial encountered no dose‐limiting toxicities, and positive HSV‐1 serology was significantly associated with enhanced survival and the clearance of CAN‐3110 from injected tumors. Post‐treatment survival, particularly in HSV‐1‐seropositive individuals, was significantly correlated with several factors: (1) alterations in tumor and peripheral blood mononuclear cell T‐cell counts and clonal diversity, (2) peripheral expansion and contraction of specific T‐cell clonotypes, and (3) tumor transcriptomic signatures indicative of immune activation. These findings provide clinical validation that intralesional oncolytic HSV therapy can potentiate anticancer immune responses, even within immunosuppressive TMEs, particularly in patients with pre‐existing serological responses to the injected virus. This approach offers a biological basis for considering oncolytic therapy in cancers that are resistant to conventional immunotherapies [128].

Ma et al. [139] developed an HSV‐1‐based OV that expresses a human IL‐15/IL‐15Rα sushi domain fusion protein, known as OV‐IL15C, in combination with off‐the‐shelf EGFR‐CAR NK cells. This combination therapy resulted in increased intracranial infiltration and activation of NK and CD8^+^ T cells, as well as improved persistence of CAR NK cells in an immunocompetent model. These findings suggest that OV‐IL15C and off‐the‐shelf EGFR‐CAR NK cells hold substantial promise as therapeutic strategies for enhancing the treatment and clinical management of GBM.

#### 5.7.3 Other OVs

Both HSV and adenovirus face important limitations in their ability to penetrate the BBB, prompting researchers to investigate alternative viral platforms and delivery methods. One such approach is the use of Toca 511, which is based on a murine leukemia virus‐derived retrovirus. Toca 511 induces direct cytotoxicity and a pro‐inflammatory state in cancer cells by converting the prodrug 5‐fluorocytosine into 5‐fluorouracil. Unfortunately, it failed to demonstrate the desired therapeutic efficacy in a phase III clinical trial (NCT02414165) [140].

Another promising strategy involves PVSRIPO, a poliovirus chimera incorporating rhinovirus elements. Administered into tumors via convection‐enhanced delivery (CED), PVSRIPO activates peripheral tumor microglia through its interaction with CD155 and subsequently recruits antigen‐presenting cells to the site, thereby enhancing the anti‐tumor immune response. This approach significantly prolongs the median overall survival and improves 2‐ and 3‐year survival rates in patients [141, 142].

Zika virus is an RNA virus that selectively targets Sox2^+^ cells with the help of integrin αvβ3, leading to increased infiltration of CD8^+^ T cells into tumor cells [143, 144]. Notably, the exploration of various viral vectors and delivery routes continues to attract attention, with particular emphasis on overcoming the BBB. Intratumoral injection and CED are the most commonly used delivery methods for these therapeutic strategies [145].

OV therapy, which involves the intratumoral infusion of genetically modified OVs, specifically targets and lyses tumor cells or stimulates immune responses. The most commonly used OVs in clinical practice include HSV and adenovirus. As research continues, these viral platforms and their delivery methods are being optimized to overcome the challenges posed by the BBB and to enhance therapeutic efficacy in the treatment of GBM.

## 6 Integrated Theranostic Approaches in GBM

### 6.1 Combination Therapies With Cellular Immune Agents

The exploration of combination therapies that integrate cellular immune agents with traditional treatments for glioma has generated great interest; this multifaceted approach holds promise for enhancing therapeutic efficacy and overcoming the limitations of existing treatments [146]. GBM are notoriously resistant to conventional interventions such as surgery, radiation, and chemotherapy. This resistance, combined with the tumor immunosuppressive microenvironment, has driven the development of innovative strategies that merge the tumor‐targeting capabilities of immune therapies with the established effects of conventional treatments.

Promising strategies in the treatment of gliomas involve the combination of CAR‐T cell therapy with radiation therapy. Radiation therapy not only elicits direct DNA damage in tumor cells but also modulates the TME in a manner that enhances the efficacy of immune therapies. Specifically, radiation can augment the expression of tumor‐associated antigens and upregulate stress signals on glioma cells, rendering them more vulnerable to recognition and destruction by CAR‐T cells. This synergistic effect has been documented in preclinical models and is currently being assessed in clinical trials [147].

Another notable combination therapy entails the use of immune checkpoint inhibitors alongside chemotherapy. Although chemotherapy primarily exerts cytotoxic effects, it can also induce immunogenic cell death, releasing tumor antigens and fostering an environment that is permissive for an immune response. When combined with immune checkpoint inhibitors, which disinhibit the immune system by blocking proteins such as PD‐1 and CTLA‐4, this strategy has demonstrated heightened anti‐tumor activity in glioma models. Clinical trials have demonstrated that this combination can lead to improved survival outcomes in patients with GBM, thus underscoring its potential as a cornerstone of future treatment protocols [148].

DC vaccines have also shown potential when used in conjunction with surgery and radiation therapy. DC vaccines function by presenting tumor antigens to T cells, thereby priming the immune system to target glioma cells. When administered following surgical resection and radiation, DC vaccines can address residual tumor cells that may have evaded initial treatment. Early‐phase clinical trials have reported instances of prolonged progression‐free survival in patients with glioma treated with this combination, suggesting a potent synergy between these approaches [149, 150].

At the mechanistic level, it has been reported that cell lines with low GD2 expression show significantly lower levels of the ganglioside synthesis enzyme ST8SIA1, also known as GD3 synthase. This enzyme is pivotal for the biosynthesis of GD2, and its downregulation creates a bottleneck that restricts the production of GD2 on the cell surface, thus contributing to resistance to anti‐GD2 therapy. Importantly, a study has unveiled a potential strategy to overcome this resistance. The pharmacological inhibition of histone‐lysine N‐methyltransferase EZH2, a crucial epigenetic regulator, reprograms mesenchymal neuroblastoma cells, leading to the re‐expression of ST8SIA1. This epigenetic rewiring restores the surface expression of GD2, thereby re‐sensitizing cells to anti‐GD2 antibodies. These findings underscore the developmental lineage of neuroblastoma cells as a critical determinant of their sensitivity to GD2‐targeted immunotherapies. Furthermore, the results suggest that EZH2 inhibitors are promising candidates for clinical testing in combination with anti‐GD2 antibodies, and may enhance therapeutic outcomes for children with neuroblastoma—particularly those who have relapsed or show resistance to current GD2‐targeted treatments [151].

Optimizing tumor‐treating fields (TTFields) parameters—including frequency, field strength, duration, and especially randomized sequence output—yields potent anti‐GBM effects by directly disrupting both tumor cell behavior and the extracellular matrix [152]. In vitro, TTFields applied at 200 kHz and up to 2.2 V/cm for 48–72 h robustly suppress the proliferation and invasiveness of U251 and patient‐derived GBM cultures, independent of their genetic backgrounds, with greater efficacy at higher “doses” and under randomized output (vs. fixed output) [153]. Mechanistically, TTFields downregulate the collagen VI alpha‐1 subunit (which is overexpressed in malignant GBM cells), thereby preventing its interaction with integrin α5 and inhibiting downstream focal adhesion kinase–paxillin–Akt signaling to impede focal adhesion dynamics. In a rat intracranial GBM model, 14 days of 200‐kHz TTFields at 2.2 V/cm reduced tumor volumes by approximately 43% (fixed) and 64% (random) and prolonged survival by 30% and 70%, respectively, with only moderate, reversible systemic changes and manageable skin reactions [154]. Collectively, these findings indicate that finely tuned, randomized‐output TTFields not only impair GBM cell growth and motility but also remodel the TME via targeted extracellular matrix modulation, supporting further clinical exploration of this non‐invasive therapy.

These examples emphasize the potential of combining cellular immune therapies with conventional glioma treatments to develop more effective and durable therapeutic strategies. As these approaches continue to be refined through clinical research, they may substantially improve outcomes for patients with this challenging and often lethal disease.

### 6.2 Real‐Time Assessment of Therapeutic Responses

Real‐time monitoring of therapeutic responses is crucial for the proficient management of glioma, allowing clinicians to promptly adjust treatment strategies in response to alterations in tumor behavior. A promising strategy for such monitoring involves the use of immune biomarkers, which provide valuable insights into treatment efficacy by reflecting the interaction between the immune system and the tumor. For example, the quantification of circulating immune cells, including T cells, NK cells, and MDSCs, can be used to assess the body's immune response to therapy. Furthermore, alterations in the expression of immune checkpoint molecules, such as PD‐1 and PD‐L1, serve as indicators of treatment effectiveness, particularly in the context of immune checkpoint inhibitors. Cytokine profiles in the blood or cerebrospinal fluid can also offer crucial information regarding the inflammatory and immune status of a patient, providing early indications of treatment success or failure [155].

#### 6.2.1 Immunotherapy Efficacy Evaluation Criteria for GBM

Based on Response Evaluation Criteria in Solid Tumors 1.1, novel terms have been proposed for evaluating the efficacy of immune therapies, such as immune complete remission, immune partial remission, immune disease stability, unconfirmed progressive disease, and confirmed progressive disease. In the case of unconfirmed progressive disease, a reconfirmation is required after 4–8 weeks to ensure assessment accuracy [156]. Additionally, the Immune‐Related Response Evaluation Criteria in Solid Tumors introduced the concept of immune best overall response, which provides a standardized approach to evaluating the response to immunotherapy over time.

The Immune‐Modified Response Evaluation Criteria in Solid Tumors standard, introduced in 2018, has built upon these concepts and aims to further refine the evaluation criteria for immune therapies. However, this standard requires continuous validation through long‐term clinical practice to ensure its robustness and reliability in various therapeutic contexts [157]. As immunotherapies become more prevalent in oncology, the ongoing refinement of these evaluation criteria will be crucial for accurately assessing treatment efficacy and guiding clinical decision‐making.

#### 6.2.2 Response Assessment in Neuro‐Oncology (RANO) 2.0

RANO 2.0 recommends using an MRI scan 21–35 days after radiotherapy as the baseline MRI for newly diagnosed gliomas; this is a departure from the previous guideline of 72 h after surgical resection. For patients with newly diagnosed GBM who have not undergone radiotherapy, the baseline MRI should be conducted after surgical resection and before initiating adjuvant therapy. Tumor progression is defined as a ≥ 25% increase in the sum of the products of the perpendicular diameters of all measurable target lesions or a ≥ 40% increase in tumor volume compared with the baseline MRI before treatment or the smallest tumor measurement after the start of treatment. Given the potential for pseudoprogression, RANO 2.0 advises reconfirming progression 3 months after radiotherapy. For recurrent GBM and World Health Organization grade 4 *IDH*‐mutant astrocytomas, RANO 2.0 recommends maintaining imaging observations for at least 3 months. Conversely, for World Health Organization grades 2 and 3 *IDH*‐mutant gliomas, imaging examinations should continue for at least 12 months. Importantly, under RANO 2.0, the worsening of clinical status or an increase in corticosteroid dosage alone does not constitute progressive disease [158].

Advanced imaging techniques play a complementary role in the theranostic approach to glioma treatment, providing non‐invasive approaches to visualize and quantify the real‐time response of a tumor to therapy. Furthermore, theranostic imaging combines diagnostic and therapeutic monitoring functions, enabling real‐time assessments of the delivery and efficacy of targeted therapies. Unlike conventional radiotherapy and chemotherapy, the success of cell immunotherapy involves various immune‐mediated effects, and the evaluation of therapy may not directly correspond to an immediate reduction in tumor volume. For cell immunotherapy, it is thus imperative to further optimize and enhance evaluation methods based on traditional criteria, incorporating the unique dynamics of immune responses.

### 6.3 Evaluation Methods for Cellular Immunotherapy

#### 6.3.1 Imaging Approaches

MRI is a versatile and powerful imaging technology that generates high‐resolution three‐dimensional images without the need for ionizing radiation [159]. The combination of PET and MRI offers substantial advantages in the identification of *IDH*‐mutant GBM [160]. Hybrid methionine–PET/MRI is particularly effective in this context [161]. Additionally, researchers have discovered that, when combined with MRI, uptake hotspots in early PET images can help to precisely locate the most malignant areas within a tumor, thereby enhancing diagnostic accuracy and improving treatment planning [161]. These advances in imaging technologies are instrumental to enhancing the precision of glioma diagnosis, treatment monitoring, and therapeutic efficacy, contributing to improved outcomes for patients with this challenging disease.

#### 6.3.2 Cellular Immunity Surveillance

The application of various methods and biomarkers for monitoring the efficacy and safety of cellular immunotherapies—particularly CAR‐T cell therapies—is crucial in clinical practice. Flow cytometry and polymerase chain reaction‐based methods are widely used to detect the dynamics and persistence of CAR‐T cells, with a focus on monitoring the absolute numbers of CD4^+^ and CD8^+^ T cells in the peripheral blood of patients [162]. Notably, peripheral blood CD8^+^CD28^+^ T‐cell levels ≥ 190/μL can predict overall and progression‐free survival, suggesting their potential as biomarkers for treatment outcomes [163]. Early memory T cells are particularly associated with the expansion and persistence of CAR‐T cells, which is crucial for the long‐term success of these therapies.

Research into the functional role of PD‐1 expression on T lymphocytes in patients with malignant glioma is ongoing. There are significant differences between PD‐1^+^ tumor‐infiltrating lymphocytes and PD‐1^+^ peripheral blood T cells; tumor‐infiltrating lymphocytes show higher levels of activation and exhaustion markers, whereas peripheral blood T cells display more memory markers and greater proliferative capacity. These differences are crucial for understanding immune responses in glioma and may influence the efficacy of PD‐1‐blocking therapies. Bioinformatic analyses have identified *SLC16A1* as a hypoxia‐associated hub gene predominantly in glioma cells, whereas monocarboxylate transporter 4 (SLC16A3) localizes to immune cells, especially TAMs and neutrophils, and is tied to neutrophil activation and pro‐metastatic chemokine expression. Both transporters closely interact with immunoinhibitory checkpoints (PD‐1/PD‐L1/PD‐L2, T‐cell immunoglobulin and mucin‐domain containing‐3) and cytokines (TGF‐β, IL‐10), and SLC16A3 levels further correlate with those of neutrophil activation markers and chemokines (e.g., CCL2, CXCL8), thereby promoting the recruitment of suppressive immune cells into the glioma microenvironment [164].

A relationship between circulating MDSCs and lymphocytopenia has also been established; monitoring MDSC levels is crucial for evaluating the efficacy of cellular immunotherapy [165]. Additionally, CXCR3^+^ monocyte expression at baseline is positively correlated with CAR‐T cell expansion, and an increase in absolute monocyte counts in circulation has been observed after CAR‐T cell administration [166].

Heterogeneity in the distribution of immune cell functional subgroups within the TME also affects clinical treatment outcomes [167]. Monitoring the infiltration sites of immune cells may provide insights into the effectiveness of cellular immunotherapies. Preliminary results from a phase I/II clinical trial evaluating a third‐generation GD2‐specific CAR construct in patients with relapsed or refractory neuroblastoma suggest its higher efficacy compared with other CAR‐T therapies. However, increased levels of polymorphonuclear MDSCs in the peripheral blood of patients who relapse or lose response to this treatment suggest that polymorphonuclear MDSCs might serve as valuable prognostic markers [168].

Dynamic monitoring of cytokines and chemokines in peripheral blood provides critical insights into the anti‐tumor effects of treatments. Imbalances in the ratio of Th1/Th2 cells are common in tumor patients, and Th1‐ and Th2‐type cytokines are useful indicators for evaluating tumor progression. Monitoring these cytokine levels can also help to identify potential toxicities, such as inflammatory responses and CRS, which are critical for managing and optimizing immunotherapy protocols [169]. The integration of these biomarkers—T‐cell dynamics, MDSC levels, and cytokine profiles—into clinical practice may therefore offer a comprehensive approach to monitoring the efficacy and safety of cellular immunotherapies in glioma and other cancers [170]. Moreover, by combining these biomarkers with advanced imaging techniques, clinicians can gain a more accurate and timely understanding of treatment responses, allowing for personalized adjustments to therapeutic strategies that enhance patient outcomes.

#### 6.3.3 EVs as Biomarkers for GBM

Increasing evidence indicates that EVs secreted from tumor cells play a pivotal role in the progression of the disease state [171, 172]. EVs, including exosomes, are secreted by a wide variety of cells and transport a diverse array of proteins, lipids, DNA, and RNA species within the body. These vesicles serve as critical mediators of intercellular communication, influencing various biological processes that contribute to tumor growth, metastasis, and TME modulation [173]. Gliomas, which constitute a substantial proportion of all primary brain tumors and the majority of brain malignancies, are particularly adept at exploiting EVs to enhance their malignant potential. Through EV release, glioma cells can alter the behavior of surrounding cells, promote angiogenesis, and evade immune surveillance, thereby facilitating overall disease progression [174].

Studies have highlighted the potential use of urinary EVs as a non‐invasive liquid biopsy approach for detecting GBM‐associated biomarkers [175, 176]. The role of the urinary system in clearing circulating EVs has led researchers to examine the proteomic profiles of urinary EVs isolated from patients with GBM and healthy controls. Using advanced mass spectrometry techniques, a large number of proteins, including known EV markers, have been identified in urinary EVs [177]. Collectively, these studies indicate the promise of urine as a source for GBM biomarker detection.

Additionally, the performance of a multiplex bead‐based flow cytometry assay, known as the EV Neuro assay, has been evaluated for detecting CNS‐derived EVs in body fluids. This assay was tested on EVs isolated from various sources, including GBM cell lines, primary human astrocytes, and blood samples from healthy controls and patients with different CNS diseases. Distinct EV profiles were reported in GBM‐derived EVs compared with human astrocytes, and specific EV populations were significantly elevated in patients with GBM. Advanced analytical techniques were able to effectively differentiate patients with GBM from healthy controls, although a similar differentiation was not achieved for patients with multiple sclerosis. However, a correlation analysis suggested a potential association between specific EV markers and disease activity in patients with multiple sclerosis [70].

Overall, these findings underscore the growing interest in EV‐based biomarkers for CNS diseases, particularly GBM. By utilizing non‐invasive methods such as urine and blood sampling, these studies provide a foundation for developing more accessible and effective diagnostic and prognostic tools, which might improve outcomes in patients with CNS diseases. However, larger cohorts are needed to allow for more robust statistical analysis and further validation of these findings.

#### 6.3.4 Next‐Generation Sequencing

Single‐cell technologies have greatly contributed to our understanding of the TME in GBM and other cancers. These technologies have revealed the intricate cellular diversity within tumors and their surrounding niche, and have been crucial for deciphering the transitions between different cell states that underlie immune adaptation over time.

One innovative technology, Zman‐seq, enables the recording of transcriptomic dynamics over time by incorporating time stamps into circulating immune cells, allowing them to be tracked within tissue over several days. When applied to GBM models, Zman‐seq has provided important insights into the transitions of immune cells within the TME, such as the dysfunction of cytotoxic NK cells and the differentiation of monocytes into immunosuppressive macrophages. Therapeutic intervention with an antagonistic anti‐triggering receptor expressed on myeloid cells 2 antibody can reshape the TME, promoting the differentiation of pro‐inflammatory macrophages; this finding underscores the potential of Zman‐seq to enhance the development of immunotherapies by precisely targeting dynamic processes within the TME [178].

Imaging mass cytometry has been used to map the immunological landscape of high‐grade gliomas and brain metastases, revealing distinct immune landscapes between primary brain tumors and brain metastases from various solid cancers. This approach has also identified specific cellular neighborhoods associated with patient survival, including a unique population of myeloperoxidase‐positive macrophages that are linked to long‐term survival in patients with GBM. These findings highlight the importance of integrating spatial context with single‐cell data to better understand cancer biology [179]. RNA sequencing on paired primary and recurrent GBM samples has revealed that GBM subtypes form a continuous and interconnected spectrum, with recurrent tumors showing mesenchymal progression and microenvironmental reorganization. Single‐cell transcriptomics and flow cytometry have also been used to track immune cell composition over time in GBM, providing a detailed view of immune cell dynamics during GBM progression [180].

The serial transplantation of GSCs into immunocompetent hosts has revealed that GSCs acquire the ability to evade immune clearance by creating an immunosuppressive TME through an epigenetic immunoediting process. This process initiates a myeloid‐affiliated transcriptional program that enhances TAM recruitment, thereby demonstrating the critical role of epigenetic immunoediting in driving immune evasion, particularly in the aggressive mesenchymal subtype of GBM [181].

Collectively, these studies highlight the transformative impact of single‐cell technologies on understanding the TME and provide new avenues for developing more effective immunotherapies for GBM and other cancers. By leveraging these technologies, researchers can gain a deeper understanding of the complex interactions within the TME and identify potential targets for therapeutic interventions, ultimately leading to improved patient outcomes [182].

#### 6.3.5 Analysis of Safety and Side Effects

CRS and ICANS are among the most common and serious complications associated with CAR‐T cell therapy. These conditions arise from the robust immune response triggered by the therapy and often require intensive management to mitigate their potentially life‐threatening effects.

##### 6.3.5.1 CRS

CRS is the most frequent and potentially severe complication of CAR‐T cell therapy. It occurs when T cells release many inflammatory cytokines and chemokines, such as IFN‐γ, TNF‐α, and GM‐CSF, into the bloodstream. Monocytes, macrophages, and DCs further exacerbate the syndrome by releasing IL‐6 and IL‐1, promoting a cascade of inflammation that can lead to fever, fatigue, and severe cardiovascular and respiratory complications [183].

##### 6.3.5.2 ICANS

ICANS was formally defined by the American Society for Transplantation and Cellular Therapy in 2019. It can occur concurrently with or following CRS. Although the exact pathophysiological mechanisms of ICANS remain unclear, it is believed that increased vascular permeability, BBB disruption, and glial cell damage contribute to its development [184]. Early symptoms of ICANS include speech and writing disorders [185].

Notably, recent findings from a phase II trial suggest that the use of the IL‐1 receptor antagonist anakinra can effectively prevent ICANS caused by CAR‐T cell therapy targeting CD19, without compromising the therapeutic efficacy of the CAR‐T cells [186].

##### 6.3.5.3 Cytopenia

Cytopenia, including anemia, thrombocytopenia, and neutropenia, is another important complication observed in patients undergoing CAR‐T cell therapy. A study of 398 patients who developed severe cytopenia within 100 days post‐therapy revealed that severe cytopenia is significantly associated with both progression‐free survival and relapse rate [187]. Early cytopenia is generally attributed to lymphocyte‐depleting chemotherapy, whereas the causes of late‐onset cytopenia remain unclear [188].

Additionally, the CAR‐HEMOTOX model has been developed to predict blood toxicity associated with CAR‐T therapy at baseline, potentially aiding in the management of cytopenia [189]. Overall, the management of these complications requires a multifaceted approach, combining supportive care, pharmacological interventions, and predictive models to optimize outcomes for patients receiving CAR‐T cell therapy.

## 7 Challenges and Prospects of Cellular Immune Diagnosis and Treatment

Cellular immune theranostics for GBM face several important challenges and limitations, arising from both technical and biological barriers [8]. These hurdles complicate the development and delivery of effective therapies targeting this aggressive brain tumor.

### 7.1 Complexity of Immune Cell Engineering

Engineering immune cells to target GBM requires highly sophisticated technologies for modifying immune cells, such as T or NK cells, to effectively recognize and attack tumor cells. Techniques such as clustered regularly interspaced short palindromic repeats (CRISPR)/CRISPR‐associated protein 9 have revolutionized gene editing, allowing for the insertion of CARs or other modifications into immune cells. However, fine‐tuning these processes is crucial for ensuring that the engineered cells not only target GBM cells with high specificity but also avoid off‐target effects that may damage healthy brain tissue [190]. Once engineered, immune cells must show controlled behavior to prevent potential adverse effects, such as excessive activation leading to CRS or ICANS. Ensuring the appropriate activation and persistence of these cells within the challenging environment of the brain is crucial.

### 7.2 Delivery to the Brain

One of the greatest barriers in GBM therapy is the BBB, which restricts the passage of therapeutic agents—including immune cells—into the brain. The BBB is a highly selective, permeable barrier that prevents most large molecules and cells from entering the CNS, making it particularly challenging to deliver engineered immune cells to GBM sites. Although innovative strategies, including intratumoral injection, CED, and techniques to transiently disrupt the BBB, have been proposed, each method presents its own set of technical hurdles and risks. For example, CED requires the placement of catheters directly into the tumor, which poses challenges in terms of precision and potential damage to surrounding healthy tissue. Additionally, disrupting the BBB can increase the risk of infections or other neurological complications, necessitating careful balancing of risks and benefits.

### 7.3 Durability and Specificity of Immune Responses

Another major challenge in GBM immunotherapy is ensuring the durability and specificity of immune responses. Once engineered immune cells reach the tumor site, their ability to effectively infiltrate the tumor and maintain functionality is often hindered by the immunosuppressive TME. The TME of GBM is highly immunosuppressive, with factors such as immunosuppressive cytokines, immune checkpoint molecules, and a lack of supportive signals impeding the persistence and functionality of immune cells. This results in immune exhaustion, where the activated immune cells lose their ability to attack the tumor effectively over time.

### 7.4 Strategies to Overcome Challenges

Researchers are currently investigating ways to improve immune cell trafficking to the brain by modifying cells with chemokine receptors or using targeted delivery systems that can navigate the BBB. Efforts to reprogram the TME to be more supportive of anti‐tumor immunity include the use of checkpoint inhibitors, TME‐modulating drugs, or genetically engineered immune cells that can resist the immunosuppressive signals within the TME. Furthermore, advanced gene editing techniques are being developed to create immune cells with enhanced specificity for GBM antigens, and strategies such as incorporating cytokine support (e.g., IL‐15) into CAR‐T or CAR‐NK cells are being explored to improve their persistence and function within the tumor environment [191]. In particular, IL‐15 has been demonstrated to enhance NK cell survival and proliferation, which may strongly boost the effectiveness of CAR‐NK therapies.

Modifying immune cells with specific chemokine receptors or the use of targeted delivery systems that can bypass or transiently disrupt the BBB are being explored as methods to improve the trafficking of immune cells to the tumor site. These approaches may allow engineered cells to more effectively reach the tumor and sustain their activity within the brain [192, 193].

### 7.5 Economic and Accessibility Challenges

In addition to the technical and biological challenges, there are important economic barriers to the widespread use of cellular immune theranostics for GBM. The high cost of developing, producing, and administering these personalized therapies is a major limitation. The sophisticated biotechnological processes involved in gene editing, cell expansion, and specialized delivery systems make the production of these therapies expensive. Moreover, the need for highly specialized infrastructure and expertise further limits their accessibility, particularly in resource‐limited settings.

To address these challenges, strategies to reduce the costs associated with cellular immune therapies must be explored. This might involve optimizing manufacturing processes for scalability, investing in automated systems to reduce labor costs, and/or fostering collaborations between public and private sectors to subsidize research and development. Additionally, implementing value‐based pricing models and expanding insurance coverage for innovative therapies may make these advanced treatments more affordable and accessible to a broader patient population. Ensuring equitable access to these therapies will be crucial for maximizing their public health impact, particularly given the high unmet need for effective treatments for GBM.

## 8 Conclusions

The exploration of cellular immune theranostics in GBM has substantially improved our comprehension of the TME and has unveiled the potential for groundbreaking therapeutic strategies. By merging immune‐based therapies with sophisticated diagnostic techniques, these approaches have shown promise for enhancing the precision and efficacy of GBM treatment. The emergence of novel immune agents, including CAR‐T cells and OVs, coupled with the use of state‐of‐the‐art imaging and biomarker technologies, underscores the transformative potential of cellular immune theranostics in GBM care. The clinical implications of these advances are profound. Cellular immune theranostics promises to not only optimize treatment outcomes through personalized approaches but also provide clinicians with real‐time assessments of therapeutic effectiveness. This capability to tailor treatments to individual tumor profiles and dynamically adjust strategies based on immediate feedback has the potential to substantially increase survival rates and enhance the quality of life for patients with GBM. Despite these encouraging developments, the domain of cellular immune theranostics for GBM remains in its nascent stages, and important work lies ahead. Ongoing research is imperative to tackle existing challenges, such as overcoming the immunosuppressive nature of the TME and improving the accessibility and cost‐effectiveness of these advanced therapies. Continuous innovation and rigorous clinical trials will be crucial for realizing the full potential of cellular immune theranostics, ultimately working toward more effective treatments and superior outcomes for patients with GBM.

## Author Contributions

**Ying Gong:** writing – original draft (lead), conceptualization (lead), funding acquisition (lead). **Wanying Lin:** writing – original draft (lead), prepared figures and tables (lead). **Xuechun Fang:** writing – original draft (equal). **Ruyi Zhang:** prepared figures and tables (equal). **Min Luo:** writing – original draft (equal), prepared figures and tables (equal). **Haoran Wu:** prepared figures and tables (equal). **Shuai Chu:** writing – original draft (equal). **Chuangkun Li:** writing – original draft (equal). **Yiming Peng:** writing – original draft (equal). **Zhiyan Piao:** writing – original draft (equal). **Siping Wu:** writing – original draft (equal). **Junhao Li:** writing – original draft (equal). **ZongZhong He:** writing – original draft (equal). **Haixia Li:** conceptualization (lead), funding acquisition (lead), supervision (lead), writing – review and editing (lead). **Hongxia Wang:** conceptualization (lead), supervision (lead), writing – review and editing (lead).

## Ethics Statement

The authors have nothing to report.

## Consent

The authors have nothing to report.

## Conflicts of Interest

The authors declare no conflicts of interest.

## Data Availability

The authors have nothing to report.

## References

[1] L. R. Schaff and I. K. Mellinghoff , “Glioblastoma and Other Primary Brain Malignancies in Adults: A Review,” Journal of the American Medical Association 329, no. 7 (2023): 574–587, 10.1001/jama.2023.0023. 36809318 PMC11445779

[2] A. Omuro , “Glioblastoma and Other Malignant Gliomas: A Clinical Review,” Journal of the American Medical Association 310, no. 17 (2013): 1842–1850, 10.1001/jama.2013.280319. 24193082

[3] Q. T. Ostrom , M. Price , C. Neff , et al., “CBTRUS Statistical Report: Primary Brain and Other Central Nervous System Tumors Diagnosed in the United States in 2016—2020,” supplement, Neuro‐Oncology 25, no. Suppl_4 (2023): iv1–iv99, 10.1093/neuonc/noad149. 37793125 PMC10550277

[4] A. C. Tan , D. M. Ashley , G. Y. López , M. Malinzak , H. S. Friedman , and M. Khasraw , “Management of Glioblastoma: State of the Art and Future Directions,” CA: A Cancer Journal for Clinicians 70, no. 4 (2020): 299–312, 10.3322/caac.21613. 32478924

[5] F. Yasinjan , Y. Xing , H. Geng , et al., “Immunotherapy: A Promising Approach for Glioma Treatment,” Frontiers in Immunology 14 (2023): 1255611, 10.3389/fimmu.2023.1255611. 37744349 PMC10512462

[6] C. Alifieris and D. T. Trafalis , “Glioblastoma Multiforme: Pathogenesis and Treatment,” Pharmacology & Therapeutics 152 (2015): 63–82, 10.1016/j.pharmthera.2015.05.005. 25944528

[7] A. Omuro , “Glioblastoma and Other Malignant Gliomas,” Journal of the American Medical Association 310, no. 17 (2013): 1842–1850, 10.1001/jama.2013.280319. 24193082

[8] S. Tanaka , D. N. Louis , W. T. Curry , T. T. Batchelor , and J. Dietrich , “Diagnostic and Therapeutic Avenues for Glioblastoma: No Longer a Dead End?,” Nature Reviews Clinical Oncology 10, no. 1 (2013): 14–26, 10.1038/nrclinonc.2012.204. 23183634

[9] C. McKinnon , M. Nandhabalan , S. A. Murray , and P. Plaha , “Glioblastoma: Clinical Presentation, Diagnosis, and Management,” BMJ 374 (2021): n1560, 10.1136/bmj.n1560. 34261630

[10] I. Noorani , P. S. Mischel , and C. Swanton , “Leveraging Extrachromosomal DNA to Fine‐Tune Trials of Targeted Therapy for Glioblastoma: Opportunities and Challenges,” Nature Reviews Clinical Oncology 19, no. 11 (2022): 733–743, 10.1038/s41571-022-00679-1. PMC13271662 36131011

[11] C. R. Goodwin and J. Laterra , “Unmasking the Multiforme in Glioblastoma,” Nature Reviews Neurology 6, no. 6 (2010): 304–305, 10.1038/nrneurol.2010.67. PMC3872833 20531430

[12] S. R. Baddam , S. Kalagara , K. Kuna , and S. Enaganti , “Recent Advancements and Theranostics Strategies in Glioblastoma Therapy,” Biomedical Materials 18, no. 5 (2023): 052007, 10.1088/1748-605x/acf0ab. 37582381

[13] M. Khasraw , Y. Fujita , C. Lee‐Chang , I. V. Balyasnikova , H. Najem , and A. B. Heimberger , “New Approaches to Glioblastoma,” Annual Review of Medicine 73 (2022): 279–292, 10.1146/annurev-med-042420-102102. PMC9479165 34665646

[14] Y. Wang , S. Li , Y. Peng , W. Ma , Y. Wang , and W. Li , “Progress in Phase III Clinical Trials of Molecular Targeted Therapy and Immunotherapy for Glioblastoma,” Cancer Innovation 2, no. 2 (2023): 114–130, 10.1002/cai2.59. 38090060 PMC10686181

[15] A. L. Hung , T. Garzon‐Muvdi , and M. Lim , “Biomarkers and Immunotherapeutic Targets in Glioblastoma,” World Neurosurgery 102 (2017): 494–506, 10.1016/j.wneu.2017.03.011. 28300714

[16] M. Touat , A. Idbaih , M. Sanson , and K. L. Ligon , “Glioblastoma Targeted Therapy: Updated Approaches From Recent Biological Insights,” Annals of Oncology 28, no. 7 (2017): 1457–1472, 10.1093/annonc/mdx106. 28863449 PMC5834086

[17] A. Bikfalvi , C. A. da Costa , T. Avril , et al., “Challenges in Glioblastoma Research: Focus on the Tumor Microenvironment,” Trends in Cancer 9, no. 1 (2023): 9–27, 10.1016/j.trecan.2022.09.005. 36400694

[18] E. Friebel , K. Kapolou , S. Unger , et al., “Single‐Cell Mapping of Human Brain Cancer Reveals Tumor‐Specific Instruction of Tissue‐Invading Leukocytes,” Cell 181, no. 7 (2020): 1626–1642.e20, 10.1016/j.cell.2020.04.055. 32470397

[19] M. González‐Tablas Pimenta , Á. Otero , D. A. Arandia Guzman , et al., “Tumor Cell and Immune Cell Profiles in Primary Human Glioblastoma: Impact on Patient Outcome,” Brain Pathology 31, no. 2 (2021): 365–380, 10.1111/bpa.12927. 33314398 PMC8018082

[20] J. Erbani , M. Boon , and L. Akkari , “Therapy‐Induced Shaping of the Glioblastoma Microenvironment: Macrophages at Play,” Seminars in Cancer Biology 86, no. 3 (2022): 41–56, 10.1016/j.semcancer.2022.05.003. 35569742

[21] J. H. Sampson , M. D. Gunn , P. E. Fecci , and D. M. Ashley , “Brain Immunology and Immunotherapy in Brain Tumours,” Nature Reviews Cancer 20, no. 1 (2020): 12–25, 10.1038/s41568-019-0224-7. 31806885 PMC7327710

[22] E. C. Cordell , M. S. Alghamri , M. G. Castro , and D. H. Gutmann , “T Lymphocytes as Dynamic Regulators of Glioma Pathobiology,” Neuro‐Oncology 24, no. 10 (2022): 1647–1657, 10.1093/neuonc/noac055. 35325210 PMC9527522

[23] K. Gousias , M. Markou , V. Arzoglou , et al., “Frequent Abnormalities of the Immune System in Gliomas and Correlation With the WHO Grading System of Malignancy,” Journal of Neuroimmunology 226, no. 1–2 (2010): 136–142, 10.1016/j.jneuroim.2010.05.027. 20605226

[24] M. K. Bhondeley , R. D. Mehra , N. K. Mehra , et al., “Imbalances in T Cell Subpopulations in Human Gliomas,” Journal of Neurosurgery 68, no. 4 (1988): 589–593, 10.3171/jns.1988.68.4.0589. 3258364

[25] T. Németh , M. Sperandio , and A. Mócsai , “Neutrophils as Emerging Therapeutic Targets,” Nature Reviews Drug Discovery 19, no. 4 (2020): 253–275, 10.1038/s41573-019-0054-z. 31969717

[26] M. Massara , P. Persico , O. Bonavita , et al., “Neutrophils in Gliomas,” Frontiers in Immunology 8 (2017): 1349, 10.3389/fimmu.2017.01349. 29123517 PMC5662581

[27] G. Wang , J. Wang , C. Niu , Y. Zhao , and P. Wu , “Neutrophils: New Critical Regulators of Glioma,” Frontiers in Immunology 13 (2022): 927233, 10.3389/fimmu.2022.927233. 35860278 PMC9289230

[28] F. Sharifzad , S. Ghavami , J. Verdi , et al., “Glioblastoma Cancer Stem Cell Biology: Potential Theranostic Targets,” Drug Resistance Updates 42 (2019): 35–45, 10.1016/j.drup.2018.03.003. 30877905

[29] A. Gomes dos Santos , R. F. de Carvalho , A. N. L. R. de Morais , et al., “Role of Neutrophil‐Lymphocyte Ratio as a Predictive Factor of Glioma Tumor Grade: A Systematic Review,” Critical Reviews in Oncology/Hematology 163 (2021): 103372, 10.1016/j.critrevonc.2021.103372. 34062242

[30] A. Bertaut , C. Truntzer , R. Madkouri , et al., “Blood Baseline Neutrophil Count Predicts Bevacizumab Efficacy in Glioblastoma,” Oncotarget 7, no. 43 (2016): 70948–70958, 10.18632/oncotarget.10898. 27487142 PMC5342600

[31] C. Sun , S. Wang , Z. Ma , et al., “Neutrophils in Glioma Microenvironment: From Immune Function to Immunotherapy,” Frontiers in Immunology 15 (2024): 1393173, 10.3389/fimmu.2024.1393173. 38779679 PMC11109384

[32] D. I. Gabrilovich and S. Nagaraj , “Myeloid‐Derived Suppressor Cells as Regulators of the Immune System,” Nature Reviews Immunology 9, no. 3 (2009): 162–174, 10.1038/nri2506. PMC2828349 19197294

[33] B. Raychaudhuri , P. Rayman , J. Ireland , et al., “Myeloid‐Derived Suppressor Cell Accumulation and Function in Patients With Newly Diagnosed Glioblastoma,” Neuro‐Oncology 13, no. 6 (2011): 591–599, 10.1093/neuonc/nor042. 21636707 PMC3107102

[34] A. Vidyarthi , T. Agnihotri , N. Khan , et al., “Predominance of M2 Macrophages in Gliomas Leads to the Suppression of Local and Systemic Immunity,” Cancer Immunology, Immunotherapy 68, no. 12 (2019): 1995–2004, 10.1007/s00262-019-02423-8. 31690954 PMC11028103

[35] D. Hambardzumyan , D. H. Gutmann , and H. Kettenmann , “The Role of Microglia and Macrophages in Glioma Maintenance and Progression,” Nature Neuroscience 19, no. 1 (2016): 20–27, 10.1038/nn.4185. 26713745 PMC4876023

[36] P. Shamshiripour , M. Rahnama , M. Nikoobakht , V. F. Rad , A.‐R. Moradi , and D. Ahmadvand , “Extracellular Vesicles Derived From Dendritic Cells Loaded With VEGF‐A siRNA and Doxorubicin Reduce Glioma Angiogenesis In Vitro,” Journal of Controlled Release 369 (2024): 128–145, 10.1016/j.jconrel.2024.03.042. 38522817

[37] M. C. Takenaka , G. Gabriely , V. Rothhammer , et al., “Control of Tumor‐Associated Macrophages and T Cells in Glioblastoma via AHR and CD39,” Nature Neuroscience 22, no. 5 (2019): 729–740, 10.1038/s41593-019-0370-y. 30962630 PMC8052632

[38] A. Heidari , P. M. Sharif , and N. Rezaei , “The Association Between Tumor‐Associated Macrophages and Glioblastoma: A Potential Target for Therapy,” Current Pharmaceutical Design 27, no. 46 (2021): 4650–4662, 10.2174/1381612827666210816114003. 34397322

[39] M. C. Morisse , S. Jouannet , M. Dominguez‐Villar , M. Sanson , and A. Idbaih , “Interactions Between Tumor‐Associated Macrophages and Tumor Cells in Glioblastoma: Unraveling Promising Targeted Therapies,” Expert Review of Neurotherapeutics 18, no. 9 (2018): 729–737, 10.1080/14737175.2018.1510321. 30099909

[40] W. Wang , T. Li , Y. Cheng , et al., “Identification of Hypoxic Macrophages in Glioblastoma With Therapeutic Potential for Vasculature Normalization,” Cancer Cell 42, no. 5 (2024): 815–832.e12, 10.1016/j.ccell.2024.03.013. 38640932

[41] G. Marelli , N. Morina , F. Portale , et al., “Lipid‐Loaded Macrophages as New Therapeutic Target in Cancer,” Journal for Immunotherapy of Cancer 10, no. 7 (2022): e004584, 10.1136/jitc-2022-004584. 35798535 PMC9263925

[42] F. Khan , L. Pang , M. Dunterman , M. S. Lesniak , A. B. Heimberger , and P. Chen , “Macrophages and Microglia in Glioblastoma: Heterogeneity, Plasticity, and Therapy,” Journal of Clinical Investigation 133, no. 1 (2023): e163446, 10.1172/jci163446. 36594466 PMC9797335

[43] M. D. Caverzán , L. Beaugé , P. M. Oliveda , B. Cesca González , E. M. Bühler , and L. E. Ibarra , “Exploring Monocytes‐Macrophages in Immune Microenvironment of Glioblastoma for the Design of Novel Therapeutic Strategies,” Brain Sciences 13, no. 4 (2023): 542, 10.3390/brainsci13040542. 37190507 PMC10136702

[44] N. Loginova , D. Aniskin , P. Timashev , I. Ulasov , and R. K. Kharwar , “GBM Immunotherapy: Macrophage Impacts,” Immunological Investigations 53, no. 5 (2024): 730–751, 10.1080/08820139.2024.2337022. 38634572

[45] S. Roesch , C. Rapp , S. Dettling , and C. Herold‐Mende , “When Immune Cells Turn Bad: Tumor‐Associated Microglia/Macrophages in Glioma,” International Journal of Molecular Sciences 19, no. 2 (2018): 436, 10.3390/ijms19020436. 29389898 PMC5855658

[46] G. Trevisi and A. Mangiola , “Current Knowledge About the Peritumoral Microenvironment in Glioblastoma,” Cancers 15, no. 22 (2023): 5460, 10.3390/cancers15225460. 38001721 PMC10670229

[47] D. Schiffer , M. Mellai , E. Bovio , I. Bisogno , C. Casalone , and L. Annovazzi , “Glioblastoma Niches: From the Concept to the Phenotypical Reality,” Neurological Sciences 39, no. 7 (2018): 1161–1168, 10.1007/s10072-018-3408-0. 29736738

[48] C. Yu‐Ju Wu , C.‐H. Chen , C.‐Y. Lin , et al., “CCL5 of Glioma‐Associated Microglia/Macrophages Regulates Glioma Migration and Invasion via Calcium‐Dependent Matrix Metalloproteinase 2,” Neuro‐Oncology 22, no. 2 (2020): 253–266, 10.1093/neuonc/noz189. 31593589 PMC7032635

[49] V. J. Cavalheiro , A. C. P. Campos , L. G. C. A. Lima , et al., “Unraveling the Peripheral and Local Role of Inflammatory Cytokines in Glioblastoma Survival,” Cytokine 161 (2023): 156059, 10.1016/j.cyto.2022.156059. 36272241

[50] A. Zisakis , C. Piperi , M. S. Themistocleous , et al., “Comparative Analysis of Peripheral and Localised Cytokine Secretion in Glioblastoma Patients,” Cytokine 39, no. 2 (2007): 99–105, 10.1016/j.cyto.2007.05.012. 17697783

[51] A. Infante Cruz , J. V. Coronel , P. Saibene Vélez , et al., “Relevance of Thymic Stromal Lymphopoietin on the Pathogenesis of Glioblastoma: Role of the Neutrophil,” Cellular and Molecular Neurobiology 44, no. 1 (2024): 31, 10.1007/s10571-024-01462-9. 38557942 PMC10984908

[52] D. Manou , M.‐A. Golfinopoulou , S. Alharbi , H. A. Alghamdi , F. M. Alzahrani , and A. D. Theocharis , “The Expression of Serglycin Is Required for Active Transforming Growth Factor β Receptor I Tumorigenic Signaling in Glioblastoma Cells and Paracrine Activation of Stromal Fibroblasts via CXCR‐2,” Biomolecules 14, no. 4 (2024): 461, 10.3390/biom14040461. 38672477 PMC11048235

[53] V. F. Zhu , J. Yang , D. G. LeBrun , and M. Li , “Understanding the Role of Cytokines in Glioblastoma Multiforme Pathogenesis,” Cancer Letters 316, no. 2 (2012): 139–150, 10.1016/j.canlet.2011.11.001. 22075379

[54] T. Hori , T. Sasayama , K. Tanaka , et al., “Tumor‐Associated Macrophage Related Interleukin‐6 in Cerebrospinal Fluid as a Prognostic Marker for Glioblastoma,” Journal of Clinical Neuroscience 68 (2019): 281–289, 10.1016/j.jocn.2019.07.020. 31327593

[55] H. Okada , G. Kohanbash , X. Zhu , et al., “Immunotherapeutic Approaches for Glioma,” Critical Reviews in Immunology 29, no. 1 (2009): 1–42, 10.1615/critrevimmunol.v29.i1.10. 19348609 PMC2713019

[56] M. L. Broekman , S. L. N. Maas , E. R. Abels , T. R. Mempel , A. M. Krichevsky , and X. O. Breakefield , “Multidimensional Communication in the Microenvirons of Glioblastoma,” Nature Reviews Neurology 14, no. 8 (2018): 482–495, 10.1038/s41582-018-0025-8. 29985475 PMC6425928

[57] A. Gieryng , D. Pszczolkowska , K. A. Walentynowicz , W. D. Rajan , and B. Kaminska , “Immune Microenvironment of Gliomas,” Laboratory Investigation 97, no. 5 (2017): 498–518, 10.1038/labinvest.2017.19. 28287634

[58] P. Y. Wen and S. Kesari , “Malignant Gliomas in Adults,” New England Journal of Medicine 359, no. 5 (2008): 492–507, 10.1056/NEJMra0708126. 18669428

[59] N. Sanai and M. S. Berger , “Surgical Oncology for Gliomas: The State of the Art,” Nature Reviews Clinical Oncology 15, no. 2 (2018): 112–125, 10.1038/nrclinonc.2017.171. 29158591

[60] J. H. Lee and C. W. Wee , “Treatment of Adult Gliomas: A Current Update,” Brain & Neurorehabilitation 15, no. 3 (2022): e24, 10.12786/bn.2022.15.e24. 36742086 PMC9833488

[61] S. Xu , L. Tang , X. Li , F. Fan , and Z. Liu , “Immunotherapy for Glioma: Current Management and Future Application,” Cancer Letters 476 (2020): 1–12, 10.1016/j.canlet.2020.02.002. 32044356

[62] O. K. Dagher , R. D. Schwab , S. K. Brookens , and A. D. Posey , “Advances in Cancer Immunotherapies,” Cell 186, no. 8 (2023): 1814–1814.e1, 10.1016/j.cell.2023.02.039. 37059073

[63] G. Oliveira and C. J. Wu , “Dynamics and Specificities of T Cells in Cancer Immunotherapy,” Nature Reviews Cancer 23, no. 5 (2023): 295–316, 10.1038/s41568-023-00560-y. 37046001 PMC10773171

[64] G. Hyman , V. Manglik , J. M. Rousch , M. Verma , D. Kinkebiel , and H. N. Banerjee , “Epigenetic Approaches in Glioblastoma Multiforme and Their Implication in Screening and Diagnosis,” Methods in Molecular Biology 1238 (2015): 511–521, 10.1007/978-1-4939-1804-1_26. 25421677

[65] G. I. Vázquez Cervantes , D. F. González Esquivel , S. Gómez‐Manzo , B. Pineda , and V. Pérez de la Cruz , “New Immunotherapeutic Approaches for Glioblastoma,” Journal of Immunology Research 2021 (2021): 3412906, 10.1155/2021/3412906. 34557553 PMC8455182

[66] E. Johnson , K. L. Dickerson , I. D. Connolly , and M. Hayden Gephart , “Single‐Cell RNA‐Sequencing in Glioma,” Current Oncology Reports 20, no. 5 (2018): 42, 10.1007/s11912-018-0673-2. 29637300 PMC8403493

[67] K. Motomura , A. Natsume , R. Watanabe , et al., “Immunohistochemical Analysis‐Based Proteomic Subclassification of Newly Diagnosed Glioblastomas,” Cancer Science 103, no. 10 (2012): 1871–1879, 10.1111/j.1349-7006.2012.02377.x. 22747609 PMC7659188

[68] V. Gilard , A. Tebani , I. Dabaj , et al., “Diagnosis and Management of Glioblastoma: A Comprehensive Perspective,” Journal of Personalized Medicine 11, no. 4 (2021): 258, 10.3390/jpm11040258. 33915852 PMC8065751

[69] I. F. Parney , J. S. Waldron , and A. T. Parsa , “Flow Cytometry and In Vitro Analysis of Human Glioma–Associated Macrophages,” Journal of Neurosurgery 110, no. 3 (2009): 572–582, 10.3171/2008.7.jns08475. 19199469 PMC3064468

[70] A. Brahmer , C. Geiß , A. Lygeraki , et al., “Assessment of Technical and Clinical Utility of a Bead‐Based Flow Cytometry Platform for Multiparametric Phenotyping of CNS‐Derived Extracellular Vesicles,” Cell Communication and Signaling 21, no. 1 (2023): 276, 10.1186/s12964-023-01308-9. 37803478 PMC10559539

[71] T. Saito , Y. Muragaki , T. Shioyama , et al., “Malignancy Index Using Intraoperative Flow Cytometry Is a Valuable Prognostic Factor for Glioblastoma Treated With Radiotherapy and Concomitant Temozolomide,” Neurosurgery 84, no. 3 (2019): 662–672, 10.1093/neuros/nyy089. 29618055

[72] A. P. Patel , I. Tirosh , J. J. Trombetta , et al., “Single‐Cell RNA‐Seq Highlights Intratumoral Heterogeneity in Primary Glioblastoma,” Science 344, no. 6190 (2014): 1396–1401, 10.1126/science.1254257. 24925914 PMC4123637

[73] S. S. Widodo , R. A. Hutchinson , Y. Fang , et al., “Toward Precision Immunotherapy Using Multiplex Immunohistochemistry and In Silico Methods to Define the Tumor Immune Microenvironment,” Cancer Immunology, Immunotherapy 70, no. 7 (2021): 1811–1820, 10.1007/s00262-020-02801-7. 33389014 PMC10991574

[74] L. R. Drake , A. T. Hillmer , and Z. Cai , “Approaches to PET Imaging of Glioblastoma,” Molecules 25, no. 3 (2020): 568, 10.3390/molecules25030568. 32012954 PMC7037643

[75] M. J. van den Bent , M. Geurts , P. J. French , et al., “Primary Brain Tumours in Adults,” Lancet 402, no. 10412 (2023): 1564–1579, 10.1016/s0140-6736(23)01054-1. 37738997

[76] J. Yue , R. Huang , Z. Lan , B. Xiao , and Z. Luo , “Abnormal Glycosylation in Glioma: Related Changes in Biology, Biomarkers and Targeted Therapy,” Biomarker Research 11, no. 1 (2023): 54, 10.1186/s40364-023-00491-8. 37231524 PMC10214721

[77] S. Sanders and W. Debinski , “Challenges to Successful Implementation of the Immune Checkpoint Inhibitors for Treatment of Glioblastoma,” International Journal of Molecular Sciences 21, no. 8 (2020): 2759, 10.3390/ijms21082759. 32316096 PMC7215941

[78] N. Majd and J. de Groot , “Challenges and Strategies for Successful Clinical Development of Immune Checkpoint Inhibitors in Glioblastoma,” Expert Opinion on Pharmacotherapy 20, no. 13 (2019): 1609–1624, 10.1080/14656566.2019.1621840. 31264484

[79] Z. Ye , X. Ai , K. Yang , et al., “Targeting Microglial Metabolic Rewiring Synergizes With Immune‐Checkpoint Blockade Therapy for Glioblastoma,” Cancer Discovery 13, no. 4 (2023): 974–1001, 10.1158/2159-8290.cd-22-0455. 36649564 PMC10073346

[80] H. Liu , Q. Zhao , L. Tan , et al., “Neutralizing IL‐8 Potentiates Immune Checkpoint Blockade Efficacy for Glioma,” Cancer Cell 41, no. 4 (2023): 693–710.e8, 10.1016/j.ccell.2023.03.004. 36963400

[81] B. Segura‐Collar , S. Hiller‐Vallina , O. de Dios , et al., “Correction to: Advanced Immunotherapies for Glioblastoma: Tumor Neoantigen Vaccines in Combination With Immunomodulators,” Acta Neuropathologica Communications 11, no. 1 (2023): 116, 10.1186/s40478-023-01600-2. 37438824 PMC10337095

[82] Z. Xiong , I. Raphael , M. Olin , H. Okada , X. Li , and G. Kohanbash , “Glioblastoma Vaccines: Past, Present, and Opportunities,” EBioMedicine 100 (2024): 104963, 10.1016/j.ebiom.2023.104963. 38183840 PMC10808938

[83] V. Dutoit , D. Migliorini , and P.‐Y. Dietrich , “Current Strategies for Vaccination in Glioblastoma,” Current Opinion in Oncology 31, no. 6 (2019): 514–521, 10.1097/cco.0000000000000575. 31403483

[84] G. P. Dunn , N. Sherpa , J. Manyanga , and T. M. Johanns , “Considerations for Personalized Neoantigen Vaccination in Malignant Glioma,” Advanced Drug Delivery Reviews 186 (2022): 114312, 10.1016/j.addr.2022.114312. 35487282

[85] M. Weller , N. Butowski , D. D. Tran , et al., “Rindopepimut With Temozolomide for Patients With Newly Diagnosed, EGFRvIII‐Expressing Glioblastoma (ACT IV): A Randomised, Double‐Blind, International Phase 3 Trial,” Lancet Oncology 18, no. 10 (2017): 1373–1385, 10.1016/S1470-2045(17)30517-X. 28844499

[86] M. Weller , P. Roth , M. Preusser , et al., “Vaccine‐Based Immunotherapeutic Approaches to Gliomas and Beyond,” Nature Reviews Neurology 13, no. 6 (2017): 363–374, 10.1038/nrneurol.2017.64. 28497804

[87] S. Wang , B. A. Castro , J. L. Katz , et al., “B Cell–Based Therapy Produces Antibodies That Inhibit Glioblastoma Growth,” Journal of Clinical Investigation 134, no. 20 (2024): e177384, 10.1172/jci177384. 39207859 PMC11473152

[88] G. Liu , N. Ma , K. Cheng , et al., “Bacteria‐Derived Nanovesicles Enhance Tumour Vaccination by Trained Immunity,” Nature Nanotechnology 19, no. 3 (2024): 387–398, 10.1038/s41565-023-01553-6. 38052943

[89] Y. Pang , X. Hou , C. Yang , Y. Liu , and G. Jiang , “Advances on Chimeric Antigen Receptor‐Modified T‐Cell Therapy for Oncotherapy,” Molecular Cancer 17, no. 1 (2018): 91, 10.1186/s12943-018-0840-y. 29769134 PMC5956614

[90] S. S. Wang , P. Bandopadhayay , and M. R. Jenkins , “Towards Immunotherapy for Pediatric Brain Tumors,” Trends in Immunology 40, no. 8 (2019): 748–761, 10.1016/j.it.2019.05.009. 31229353

[91] M. R. Jenkins and K. J. Drummond , “CAR T‐Cell Therapy for Glioblastoma,” New England Journal of Medicine 390, no. 14 (2024): 1329–1332, 10.1056/nejme2401307. 38598802

[92] K. Qian , G. Li , S. Zhang , et al., “CAR‐T‐Cell Products in Solid Tumors: Progress, Challenges, and Strategies,” Interdisciplinary Medicine 2, no. 2 (2024): e20230047, 10.1002/inmd.20230047.

[93] B. Dewdney , M. R. Jenkins , S. A. Best , et al., “From Signalling Pathways to Targeted Therapies: Unravelling Glioblastoma's Secrets and Harnessing Two Decades of Progress,” Signal Transduction and Targeted Therapy 8, no. 1 (2023): 400, 10.1038/s41392-023-01637-8. 37857607 PMC10587102

[94] M. Kilian , R. Sheinin , C. L. Tan , et al., “MHC Class II‐Restricted Antigen Presentation Is Required to Prevent Dysfunction of Cytotoxic T Cells by Blood‐Borne Myeloids in Brain Tumors,” Cancer Cell 41, no. 2 (2023): 235–251.e9, 10.1016/j.ccell.2022.12.007. 36638785

[95] F. Del Bufalo , B. De Angelis , I. Caruana , et al., “GD2‐CART01 for Relapsed or Refractory High‐Risk Neuroblastoma,” New England Journal of Medicine 388, no. 14 (2023): 1284–1295, 10.1056/nejmoa2210859. 37018492

[96] R. G. Majzner , S. Ramakrishna , K. W. Yeom , et al., “GD2‐CAR T Cell Therapy for H3K27M‐Mutated Diffuse Midline Gliomas,” Nature 603, no. 7903 (2022): 934–941, 10.1038/s41586-022-04489-4. 35130560 PMC8967714

[97] D. Alizadeh , R. A. Wong , S. Gholamin , et al., “IFNγ Is Critical for CAR T Cell–Mediated Myeloid Activation and Induction of Endogenous Immunity,” Cancer Discovery 11, no. 9 (2021): 2248–2265, 10.1158/2159-8290.cd-20-1661. 33837065 PMC8561746

[98] H. Meister , T. Look , P. Roth , et al., “Multifunctional MRNA‐Based CAR T Cells Display Promising Antitumor Activity Against Glioblastoma,” Clinical Cancer Research 28, no. 21 (2022): 4747–4756, 10.1158/1078-0432.ccr-21-4384. 36037304

[99] G. Wang , Z. Zhang , K. Zhong , et al., “CXCL11‐Armed Oncolytic Adenoviruses Enhance CAR‐T Cell Therapeutic Efficacy and Reprogram Tumor Microenvironment in Glioblastoma,” Molecular Therapy 31, no. 1 (2023): 134–153, 10.1016/j.ymthe.2022.08.021. 36056553 PMC9840126

[100] T. Weiss , M. Weller , M. Guckenberger , C. L. Sentman , and P. Roth , “NKG2D‐Based CAR T Cells and Radiotherapy Exert Synergistic Efficacy in Glioblastoma,” Cancer Research 78, no. 4 (2018): 1031–1043, 10.1158/0008-5472.can-17-1788. 29222400

[101] D. Yang , B. Sun , H. Dai , et al., “T Cells Expressing NKG2D Chimeric Antigen Receptors Efficiently Eliminate Glioblastoma and Cancer Stem Cells,” Journal for Immunotherapy of Cancer 7, no. 1 (2019): 171, 10.1186/s40425-019-0642-9. 31288857 PMC6617951

[102] Y. Zhang , J. Xie , H. Wu , et al., “NK Cell Based Immunotherapy Against Oral Squamous Cell Carcinoma,” Frontiers in Immunology 15 (2024): 1440764, 10.3389/fimmu.2024.1440764. 39192980 PMC11347299

[103] Y. Gong , W. T. V. Germeraad , X. Zhang , et al., “NKG2A Genetic Deletion Promotes Human Primary NK Cell Anti‐Tumor Responses Better Than an Anti‐NKG2A Monoclonal Antibody,” Molecular Therapy 32, no. 8 (2024): 2711–2727, 10.1016/j.ymthe.2024.06.034. 38943249 PMC11405175

[104] M. I. Strecker , K. Wlotzka , F. Strassheimer , et al., “AAV‐Mediated Gene Transfer of a Checkpoint Inhibitor in Combination With HER2‐Targeted CAR‐NK Cells as Experimental Therapy for Glioblastoma,” Oncoimmunology 11, no. 1 (2022): 2127508, 10.1080/2162402x.2022.2127508. 36249274 PMC9559045

[105] D. Kong , D. Kwon , B. Moon , et al., “CD19 CAR‐Expressing IPSC‐Derived NK Cells Effectively Enhance Migration and Cytotoxicity Into Glioblastoma by Targeting to the Pericytes in Tumor Microenvironment,” Biomedicine & Pharmacotherapy = Biomedecine & Pharmacotherapie 174 (2024): 116436, 10.1016/j.biopha.2024.116436. 38508081

[106] K. Chaudhry , A. Geiger , E. Dowlati , et al., “Co‐Transducing B7H3 CAR‐NK Cells With the DNR Preserves Their Cytolytic Function Against GBM in the Presence of Exogenous TGF‐Β,” Molecular Therapy. Methods & Clinical Development 27 (2022): 415–430, 10.1016/j.omtm.2022.10.010. 36381305 PMC9661497

[107] M. C. Burger , M.‐T. Forster , A. Romanski , et al., “Intracranial Injection of Natural Killer Cells Engineered With a HER2‐Targeted Chimeric Antigen Receptor in Patients With Recurrent Glioblastoma,” Neuro‐Oncology 25, no. 11 (2023): 2058–2071, 10.1093/neuonc/noad087. 37148198 PMC10628939

[108] A. Heczey , X. Xu , A. N. Courtney , et al., “Anti‐GD2 CAR‐NKT Cells in Relapsed or Refractory Neuroblastoma: Updated Phase 1 Trial Interim Results,” Nature Medicine 29, no. 6 (2023): 1379–1388, 10.1038/s41591-023-02363-y. 37188782

[109] A. Heczey , A. N. Courtney , A. Montalbano , et al., “Anti‐GD2 CAR‐NKT Cells in Patients With Relapsed or Refractory Neuroblastoma: An Interim Analysis,” Nature Medicine 26, no. 11 (2020): 1686–1690, 10.1038/s41591-020-1074-2. 33046868

[110] G. Wang , K. Zhong , Z. Wang , et al., “Tumor‐Associated Microglia and Macrophages in Glioblastoma: From Basic Insights to Therapeutic Opportunities,” Frontiers in Immunology 13 (2022): 964898, 10.3389/fimmu.2022.964898. 35967394 PMC9363573

[111] S. M. Pyonteck , L. Akkari , A. J. Schuhmacher , et al., “CSF‐1R Inhibition Alters Macrophage Polarization and Blocks Glioma Progression,” Nature Medicine 19, no. 10 (2013): 1264–1272, 10.1038/nm.3337. PMC3840724 24056773

[112] N. Butowski , H. Colman , J. F. De Groot , et al., “Orally Administered Colony Stimulating Factor 1 Receptor Inhibitor PLX3397 in Recurrent Glioblastoma: An Ivy Foundation Early Phase Clinical Trials Consortium Phase II Study,” Neuro‐Oncology 18, no. 4 (2016): 557–564, 10.1093/neuonc/nov245. 26449250 PMC4799682

[113] V. Fermi , R. Warta , A. Wöllner , et al., “Effective Reprogramming of Patient‐Derived M2‐Polarized Glioblastoma‐Associated Microglia/Macrophages by Treatment With GW2580,” Clinical Cancer Research 29, no. 22 (2023): 4685–4697, 10.1158/1078-0432.ccr-23-0576. 37682326

[114] K. Yang , W. Han , X. Jiang , et al., “Zinc Cyclic Di‐AMP Nanoparticles Target and Suppress Tumours via Endothelial STING Activation and Tumour‐Associated Macrophage Reinvigoration,” Nature Nanotechnology 17, no. 12 (2022): 1322–1331, 10.1038/s41565-022-01225-x. 36302963

[115] S. Xu , J. Wei , F. Wang , et al., “Effect of miR‐142‐3p on the M2 Macrophage and Therapeutic Efficacy Against Murine Glioblastoma,” JNCI: Journal of the National Cancer Institute 106, no. 8 (2014): dju162, 10.1093/jnci/dju162. 24974128 PMC4271080

[116] C. Chen , W. Jing , Y. Chen , et al., “Intracavity Generation of Glioma Stem Cell–Specific CAR Macrophages Primes Locoregional Immunity for Postoperative Glioblastoma Therapy,” Science Translational Medicine 14, no. 656 (2022): eabn1128, 10.1126/scitranslmed.abn1128. 35921473

[117] J. H. Choe , P. B. Watchmaker , M. S. Simic , et al., “SynNotch‐CAR T Cells Overcome Challenges of Specificity, Heterogeneity, and Persistence in Treating Glioblastoma,” Science Translational Medicine 13, no. 591 (2021): eabe7378, 10.1126/scitranslmed.abe7378. 33910979 PMC8362330

[118] B. D. Choi , E. R. Gerstner , M. J. Frigault , et al., “Intraventricular CARv3‐TEAM‐E T Cells in Recurrent Glioblastoma,” New England Journal of Medicine 390, no. 14 (2024): 1290–1298, 10.1056/nejmoa2314390. 38477966 PMC11162836

[119] C. E. Brown , J. C. Hibbard , D. Alizadeh , et al., “Locoregional Delivery of IL‐13Rα2‐Targeting CAR‐T Cells in Recurrent High‐Grade Glioma: A Phase 1 Trial,” Nature Medicine 30, no. 4 (2024): 1001–1012, 10.1038/s41591-024-02875-1. PMC11031404 38454126

[120] S. J. Bagley , M. Logun , J. A. Fraietta , et al., “Intrathecal Bivalent CAR T Cells Targeting EGFR and IL13Rα2 in Recurrent Glioblastoma: Phase 1 Trial Interim Results,” Nature Medicine 30, no. 5 (2024): 1320–1329, 10.1038/s41591-024-02893-z. PMC13123313 38480922

[121] C. Flüh , G. Chitadze , V. Adamski , et al., “NKG2D Ligands in Glioma Stem‐Like Cells: Expression In Situ and In Vitro,” Histochemistry and Cell Biology 149, no. 3 (2018): 219–233, 10.1007/s00418-018-1633-5. 29356965

[122] N. A. Vitanza , A. L. Wilson , W. Huang , et al., “Intraventricular B7‐H3 CAR T Cells for Diffuse Intrinsic Pontine Glioma: Preliminary First‐in‐Human Bioactivity and Safety,” Cancer Discovery 13, no. 1 (2023): 114–131, 10.1158/2159-8290.cd-22-0750. 36259971 PMC9827115

[123] K. Straathof , B. Flutter , R. Wallace , et al., “Antitumor Activity Without On‐Target Off‐Tumor Toxicity of GD2–Chimeric Antigen Receptor T Cells in Patients With Neuroblastoma,” Science Translational Medicine 12, no. 571 (2020): eabd6169, 10.1126/scitranslmed.abd6169. 33239386

[124] Z. Liu , J. Zhou , X. Yang , et al., “Safety and Antitumor Activity of GD2‐Specific 4SCAR‐T Cells in Patients With Glioblastoma,” Molecular Cancer 22, no. 1 (2023): 3, 10.1186/s12943-022-01711-9. 36617554 PMC9827625

[125] M. Yankelevich , A. Thakur , S. Modak , et al., “Targeting Refractory/Recurrent Neuroblastoma and Osteosarcoma With Anti‐CD3 × Anti‐GD2 Bispecific Antibody Armed T Cells,” Journal for Immunotherapy of Cancer 12, no. 3 (2024): e008744, 10.1136/jitc-2023-008744. 38519053 PMC10961524

[126] J. Deshane , C. C. Garner , and H. Sontheimer , “Chlorotoxin Inhibits Glioma Cell Invasion via Matrix Metalloproteinase‐2,” Journal of Biological Chemistry 278, no. 6 (2003): 4135–4144, 10.1074/jbc.M205662200. 12454020

[127] D. Wang , R. Starr , W.‐C. Chang , et al., “Chlorotoxin‐Directed CAR T Cells for Specific and Effective Targeting of Glioblastoma,” Science Translational Medicine 12, no. 533 (2020): eaaw2672, 10.1126/scitranslmed.aaw2672. 32132216 PMC7500824

[128] A. L. Ling , I. H. Solomon , A. M. Landivar , et al., “Clinical Trial Links Oncolytic Immunoactivation to Survival in Glioblastoma,” Nature 623, no. 7985 (2023): 157–166, 10.1038/s41586-023-06623-2. 37853118 PMC10620094

[129] Y. R. Suryawanshi and A. J. Schulze , “Oncolytic Viruses for Malignant Glioma: On the Verge of Success?,” Viruses 13, no. 7 (2021): 1294, 10.3390/v13071294. 34372501 PMC8310195

[130] J. Fueyo , R. Alemany , C. Gomez‐Manzano , et al., “Preclinical Characterization of the Antiglioma Activity of a Tropism‐Enhanced Adenovirus Targeted to the Retinoblastoma Pathway,” JNCI: Journal of the National Cancer Institute 95, no. 9 (2003): 652–660, 10.1093/jnci/95.9.652. 12734316

[131] F. F. Lang , C. Conrad , C. Gomez‐Manzano , et al., “Phase I Study of DNX‐2401 (Delta‐24‐RGD) Oncolytic Adenovirus: Replication and Immunotherapeutic Effects in Recurrent Malignant Glioma,” Journal of Clinical Oncology 36, no. 14 (2018): 1419–1427, 10.1200/jco.2017.75.8219. 29432077 PMC6075856

[132] F. Nassiri , V. Patil , L. S. Yefet , et al., “Oncolytic DNX‐2401 Virotherapy Plus Pembrolizumab in Recurrent Glioblastoma: A Phase 1/2 Trial,” Nature Medicine 29, no. 6 (2023): 1370–1378, 10.1038/s41591-023-02347-y. PMC10287560 37188783

[133] M. L. Varela , A. Comba , S. M. Faisal , et al., “Gene Therapy for High Grade Glioma: The Clinical Experience,” Expert Opinion on Biological Therapy 23, no. 2 (2023): 145–161, 10.1080/14712598.2022.2157718. 36510843 PMC9998375

[134] Y. Umemura , D. Orringer , L. Junck , et al., “Combined Cytotoxic and Immune‐Stimulatory Gene Therapy for Primary Adult High‐Grade Glioma: A Phase 1, First‐in‐Human Trial,” Lancet Oncology 24, no. 9 (2023): 1042–1052, 10.1016/S1470-2045(23)00347-9. 37657463

[135] M. Ramachandran , A. Vaccaro , T. van de Walle , et al., “Tailoring Vascular Phenotype Through AAV Therapy Promotes Anti‐Tumor Immunity in Glioma,” Cancer Cell 41, no. 6 (2023): 1134–1151.e10, 10.1016/j.ccell.2023.04.010. 37172581

[136] J. Gállego Pérez‐Larraya , M. Garcia‐Moure , S. Labiano , et al., “Oncolytic DNX‐2401 Virus for Pediatric Diffuse Intrinsic Pontine Glioma,” New England Journal of Medicine 386, no. 26 (2022): 2471–2481, 10.1056/nejmoa2202028. 35767439

[137] T. Todo , H. Ito , Y. Ino , et al., “Intratumoral Oncolytic Herpes Virus G47∆ for Residual or Recurrent Glioblastoma: A Phase 2 Trial,” Nature Medicine 28, no. 8 (2022): 1630–1639, 10.1038/s41591-022-01897-x. PMC9388376 35864254

[138] J. D. Christie and E. A. Chiocca , “Treat and Repeat: Oncolytic Virus Therapy for Brain Cancer,” Nature Medicine 28, no. 8 (2022): 1540–1542, 10.1038/s41591-022-01901-4. 35864255

[139] R. Ma , T. Lu , Z. Li , et al., “An Oncolytic Virus Expressing IL15/IL15Rα Combined With Off‐the‐Shelf EGFR‐CAR NK Cells Targets Glioblastoma,” Cancer Research 81, no. 13 (2021): 3635–3648, 10.1158/0008-5472.can-21-0035. 34006525 PMC8562586

[140] T. F. Cloughesy , K. Petrecca , T. Walbert , et al., “Effect of Vocimagene Amiretrorepvec in Combination With Flucytosine vs Standard of Care on Survival Following Tumor Resection in Patients With Recurrent High‐Grade Glioma,” JAMA Oncology 6, no. 12 (2020): 1939, 10.1001/jamaoncol.2020.3161. 33119048 PMC7596685

[141] A. Desjardins , M. Gromeier , J. E. Herndon , et al., “Recurrent Glioblastoma Treated With Recombinant Poliovirus,” New England Journal of Medicine 379, no. 2 (2018): 150–161, 10.1056/nejmoa1716435. 29943666 PMC6065102

[142] Y. Yang , M. C. Brown , G. Zhang , et al., “Polio Virotherapy Targets the Malignant Glioma Myeloid Infiltrate With Diffuse Microglia Activation Engulfing the CNS,” Neuro‐Oncology 25, no. 9 (2023): 1631–1643, 10.1093/neuonc/noad052. 36864784 PMC10479910

[143] Z. Zhu , P. Mesci , J. A. Bernatchez , et al., “Zika Virus Targets Glioblastoma Stem Cells Through a SOX2‐Integrin Αvβ5 Axis,” Cell Stem Cell 26, no. 2 (2020): 187–204.e10, 10.1016/j.stem.2019.11.016. 31956038 PMC9628766

[144] S. Nair , L. Mazzoccoli , A. Jash , et al., “Zika Virus Oncolytic Activity Requires CD8^+^ T Cells and Is Boosted by Immune Checkpoint Blockade,” JCI Insight 6, no. 1 (2021): e144619, 10.1172/jci.insight.144619. 33232299 PMC7821591

[145] M. Hu , X. Liao , Y. Tao , and Y. Chen , “Advances in Oncolytic Herpes Simplex Virus and Adenovirus Therapy for Recurrent Glioma,” Frontiers in Immunology 14 (2023): 1285113, 10.3389/fimmu.2023.1285113. 38022620 PMC10652401

[146] M. Zhao , D. van Straten , M. L. D. Broekman , V. Préat , and R. M. Schiffelers , “Nanocarrier‐Based Drug Combination Therapy for Glioblastoma,” Theranostics 10, no. 3 (2020): 1355–1372, 10.7150/thno.38147. 31938069 PMC6956816

[147] Y. Yang , S. Badeti , H.‐C. Tseng , et al., “Superior Expansion and Cytotoxicity of Human Primary NK and CAR‐NK Cells From Various Sources via Enriched Metabolic Pathways,” Molecular Therapy. Methods & Clinical Development 18 (2020): 428–445, 10.1016/j.omtm.2020.06.014. 32695845 PMC7364029

[148] J. M. Erickson , N. Tokarek , W. Ke , and A. Swartz , “A Randomized Controlled Trial of a Physical Activity Intervention for Self‐Management of Fatigue in Adolescents and Young Adults With Cancer,” Cancer Nursing 44, no. 4 (2021): 263–271, 10.1097/ncc.0000000000000834. 32568808 PMC7744366

[149] L. Li , J. Zhou , X. Dong , Q. Liao , D. Zhou , and Y. Zhou , “Dendritic Cell Vaccines for Glioblastoma Fail to Complete Clinical Translation: Bottlenecks and Potential Countermeasures,” International Immunopharmacology 109 (2022): 108929, 10.1016/j.intimp.2022.108929. 35700581

[150] K. M. Hotchkiss , K. A. Batich , A. Mohan , R. Rahman , S. Piantadosi , and M. Khasraw , “Dendritic Cell Vaccine Trials in Gliomas: Untangling the Lines,” Neuro‐Oncology 25, no. 10 (2023): 1752–1762, 10.1093/neuonc/noad088. 37289203 PMC10547519

[151] N. W. Mabe , M. Huang , G. N. Dalton , et al., “Transition to a Mesenchymal State in Neuroblastoma Confers Resistance to Anti‐GD2 Antibody via Reduced Expression of ST8SIA1,” Nature Cancer 3, no. 8 (2022): 976–993, 10.1038/s43018-022-00405-x. 35817829 PMC10071839

[152] J. Lan , Y. Liu , J. Chen , et al., “Advanced Tumor Electric Fields Therapy: A Review of Innovative Research and Development and Prospect of Application in Glioblastoma,” CNS Neuroscience & Therapeutics 30, no. 5 (2024): e14720, 10.1111/cns.14720. 38715344 PMC11077002

[153] H. Wu , L. Yang , H. Liu , et al., “Exploring the Efficacy of Tumor Electric Field Therapy Against Glioblastoma: An In Vivo and In Vitro Study,” CNS Neuroscience & Therapeutics 27, no. 12 (2021): 1587–1604, 10.1111/cns.13750. 34710276 PMC8611775

[154] J. Chen , Y. Liu , J. Lan , et al., “Identification and Validation of COL6A1 as a Novel Target for Tumor Electric Field Therapy in Glioblastoma,” CNS Neuroscience & Therapeutics 30, no. 6 (2024): e14802, 10.1111/cns.14802. 38887185 PMC11183175

[155] A. Mohammadzadeh , I. A. Rad , and B. Ahmadi‐Salmasi , “CTLA‐4, PD‐1 and TIM‐3 Expression Predominantly Downregulated in MS Patients,” Journal of Neuroimmunology 323 (2018): 105–108, 10.1016/j.jneuroim.2018.08.004. 30196822

[156] L. Seymour , J. Bogaerts , A. Perrone , et al., “IRECIST: Guidelines for Response Criteria for Use in Trials Testing Immunotherapeutics,” Lancet Oncology 18, no. 3 (2017): e143–e152, 10.1016/S1470-2045(17)30074-8. 28271869 PMC5648544

[157] F. S. Hodi , M. Ballinger , B. Lyons , et al., “Immune‐Modified Response Evaluation Criteria in Solid Tumors (imRECIST): Refining Guidelines to Assess the Clinical Benefit of Cancer Immunotherapy,” Journal of Clinical Oncology 36, no. 9 (2018): 850–858, 10.1200/jco.2017.75.1644. 29341833

[158] P. Y. Wen , M. van den Bent , G. Youssef , et al., “RANO 2.0: Update to the Response Assessment in Neuro‐Oncology Criteria for High‐ and Low‐Grade Gliomas in Adults,” Journal of Clinical Oncology 41, no. 33 (2023): 5187–5199, 10.1200/jco.23.01059. 37774317 PMC10860967

[159] L. Liu , C. W. Yoon , Z. Yuan , et al., “Cellular and Molecular Imaging of CAR‐T Cell‐Based Immunotherapy,” Advanced Drug Delivery Reviews 203 (2023): 115135, 10.1016/j.addr.2023.115135. 37931847 PMC11052581

[160] F.‐Y. Chiu and Y. Yen , “Imaging Biomarkers for Clinical Applications in Neuro‐Oncology: Current Status and Future Perspectives,” Biomarker Research 11, no. 1 (2023): 35, 10.1186/s40364-023-00476-7. 36991494 PMC10053808

[161] M. Harat , J. Rakowska , M. Harat , et al., “Combining Amino Acid PET and MRI Imaging Increases Accuracy to Define Malignant Areas in Adult Glioma,” Nature Communications 14, no. 1 (2023): 4572, 10.1038/s41467-023-39731-8. PMC10387066 37516762

[162] U. Blache , R. Weiss , A. Boldt , et al., “Advanced Flow Cytometry Assays for Immune Monitoring of CAR‐T Cell Applications,” Frontiers in Immunology 12 (2021): 658314, 10.3389/fimmu.2021.658314. 34012442 PMC8127837

[163] R. Geng , H. Tang , T. You , et al., “Peripheral CD8^+^CD28^+^ T Lymphocytes Predict the Efficacy and Safety of PD‐1/PD‐L1 Inhibitors in Cancer Patients,” Frontiers in Immunology 14 (2023): 1125876, 10.3389/fimmu.2023.1125876. 36969245 PMC10038730

[164] T. Zhu , X. Ge , S. Gong , et al., “Prognostic Value of Lactate Transporter SLC16A1 and SLC16A3 as Oncoimmunological Biomarkers Associating Tumor Metabolism and Immune Evasion in Glioma,” Cancer Innovation 1, no. 3 (2022): 229–239, 10.1002/cai2.32. 38089757 PMC10686114

[165] S. Ghosh , J. Huang , M. Inkman , et al., “Radiation‐Induced Circulating Myeloid‐Derived Suppressor Cells Induce Systemic Lymphopenia After Chemoradiotherapy in Patients With Glioblastoma,” Science Translational Medicine 15, no. 680 (2023): eabn6758, 10.1126/scitranslmed.abn6758. 36696484 PMC10501302

[166] S. Kaczanowska , T. Murty , A. Alimadadi , et al., “Immune Determinants of CAR‐T Cell Expansion in Solid Tumor Patients Receiving GD2 CAR‐T Cell Therapy,” Cancer Cell 42, no. 1 (2024): 35–51.e8, 10.1016/j.ccell.2023.11.011. 38134936 PMC10947809

[167] W. H. Fridman , F. Pagès , C. Sautès‐Fridman , and J. Galon , “The Immune Contexture in Human Tumours: Impact on Clinical Outcome,” Nature Reviews Cancer 12, no. 4 (2012): 298–306, 10.1038/nrc3245. 22419253

[168] N. Tumino , G. Weber , F. Besi , et al., “Polymorphonuclear Myeloid‐Derived Suppressor Cells Impair the Anti‐Tumor Efficacy of GD2.CAR T‐Cells in Patients With Neuroblastoma,” Journal of Hematology & Oncology 14, no. 1 (2021): 191, 10.1186/s13045-021-01193-0. 34772439 PMC8588686

[169] W. P. Accomando , A. R. Rao , D. J. Hogan , et al., “Molecular and Immunologic Signatures Are Related to Clinical Benefit From Treatment With Vocimagene Amiretrorepvec (*Toca* 511) and 5‐Fluorocytosine (*Toca* FC) in Patients With Glioma,” Clinical Cancer Research 26, no. 23 (2020): 6176–6186, 10.1158/1078-0432.ccr-20-0536. 32816892

[170] F. Klemm , R. R. Maas , R. L. Bowman , et al., “Interrogation of the Microenvironmental Landscape in Brain Tumors Reveals Disease‐Specific Alterations of Immune Cells,” Cell 181, no. 7 (2020): 1643–1660.e17, 10.1016/j.cell.2020.05.007. 32470396 PMC8558904

[171] J. Dai , Y. Jiang , H. Hu , S. Zhang , and Y. Chen , “Extracellular Vesicles as Modulators of Glioblastoma Progression and Tumor Microenvironment,” Pathology and Oncology Research 30 (2024): 1611549, 10.3389/pore.2024.1611549. 38379858 PMC10876843

[172] Y. Huang , T. Arab , A. E. Russell , et al., “Toward a Human Brain Extracellular Vesicle Atlas: Characteristics of Extracellular Vesicles From Different Brain Regions, Including Small RNA and Protein Profiles,” Interdisciplinary Medicine 1, no. 4 (2023): e20230016, 10.1002/inmd.20230016. 38089920 PMC10712435

[173] K. Mahmoudi , A. Ezrin , and C. Hadjipanayis , “Small Extracellular Vesicles as Tumor Biomarkers for Glioblastoma,” Molecular Aspects of Medicine 45 (2015): 97–102, 10.1016/j.mam.2015.06.008. 26118341

[174] B. Basu and M. K. Ghosh , “Extracellular Vesicles in Glioma: From Diagnosis to Therapy,” BioEssays 41, no. 7 (2019): 1800245, 10.1002/bies.201800245. 31188499

[175] M. N. Russo , L. A. Whaley , E. S. Norton , N. Zarco , and H. Guerrero‐Cázares , “Extracellular Vesicles in the Glioblastoma Microenvironment: A Diagnostic and Therapeutic Perspective,” Molecular Aspects of Medicine 91 (2023): 101167, 10.1016/j.mam.2022.101167. 36577547 PMC10073317

[176] L. Zhang , W. Ma , X. Gan , et al., “Adequate Enrichment of Extracellular Vesicles in Laboratory Medicine,” Interdisciplinary Medicine 1, no. 3 (2023): e20220003, 10.1002/inmd.20220003.

[177] S. M. Hallal , Á. Tűzesi , L. A. Sida , et al., “Glioblastoma Biomarkers in Urinary Extracellular Vesicles Reveal the Potential for a ‘Liquid Gold’ Biopsy,” British Journal of Cancer 130, no. 5 (2024): 836–851, 10.1038/s41416-023-02548-9. 38212481 PMC10912426

[178] D. Kirschenbaum , K. Xie , F. Ingelfinger , et al., “Time‐Resolved Single‐Cell Transcriptomics Defines Immune Trajectories in Glioblastoma,” Cell 187, no. 1 (2024): 149–165.e23, 10.1016/j.cell.2023.11.032. 38134933

[179] E. Karimi , M. W. Yu , S. M. Maritan , et al., “Single‐Cell Spatial Immune Landscapes of Primary and Metastatic Brain Tumours,” Nature 614, no. 7948 (2023): 555–563, 10.1038/s41586-022-05680-3. 36725935 PMC9931580

[180] Y. Hoogstrate , K. Draaisma , S. A. Ghisai , et al., “Transcriptome Analysis Reveals Tumor Microenvironment Changes in Glioblastoma,” Cancer Cell 41, no. 4 (2023): 678–692.e7, 10.1016/j.ccell.2023.02.019. 36898379

[181] A. T. Yeo , S. Rawal , B. Delcuze , et al., “Single‐Cell RNA Sequencing Reveals Evolution of Immune Landscape During Glioblastoma Progression,” Nature Immunology 23, no. 6 (2022): 971–984, 10.1038/s41590-022-01215-0. 35624211 PMC9174057

[182] E. Gangoso , B. Southgate , L. Bradley , et al., “Glioblastomas Acquire Myeloid‐Affiliated Transcriptional Programs via Epigenetic Immunoediting to Elicit Immune Evasion,” Cell 184, no. 9 (2021): 2454–2470.e26, 10.1016/j.cell.2021.03.023. 33857425 PMC8099351

[183] M.‐L. Schubert , M. Schmitt , L. Wang , et al., “Side‐Effect Management of Chimeric Antigen Receptor (CAR) T‐Cell Therapy,” Annals of Oncology 32, no. 1 (2021): 34–48, 10.1016/j.annonc.2020.10.478. 33098993

[184] E. C. Morris , S. S. Neelapu , T. Giavridis , and M. Sadelain , “Cytokine Release Syndrome and Associated Neurotoxicity in Cancer Immunotherapy,” Nature Reviews Immunology 22, no. 2 (2022): 85–96, 10.1038/s41577-021-00547-6. PMC8127450 34002066

[185] S. S. Neelapu , S. Tummala , P. Kebriaei , et al., “Chimeric Antigen Receptor T‐Cell Therapy: Assessment and Management of Toxicities,” Nature Reviews Clinical Oncology 15, no. 1 (2018): 47–62, 10.1038/nrclinonc.2017.148. PMC6733403 28925994

[186] J. H. Park , K. Nath , S. M. Devlin , et al., “CD19 CAR T‐Cell Therapy and Prophylactic Anakinra in Relapsed or Refractory Lymphoma: Phase 2 Trial Interim Results,” Nature Medicine 29, no. 7 (2023): 1710–1717, 10.1038/s41591-023-02404-6. PMC11462637 37400640

[187] O. Penack , C. Peczynski , C. Koenecke , et al., “Severe Cytopenia After CD19 CAR T‐Cell Therapy: A Retrospective Study From the EBMT Transplant Complications Working Party,” Journal for Immunotherapy of Cancer 11, no. 4 (2023): e006406, 10.1136/jitc-2022-006406. 37072350 PMC10124318

[188] S. Fried , A. Avigdor , B. Bielorai , et al., “Early and Late Hematologic Toxicity Following CD19 CAR‐T Cells,” Bone Marrow Transplantation 54, no. 10 (2019): 1643–1650, 10.1038/s41409-019-0487-3. 30809033

[189] K. Rejeski , A. Perez , P. Sesques , et al., “CAR‐HEMATOTOX: A Model for CAR T‐Cell–Related Hematologic Toxicity in Relapsed/Refractory Large B‐Cell Lymphoma,” Blood 138, no. 24 (2021): 2499–2513, 10.1182/blood.2020010543. 34166502 PMC8893508

[190] N. Laperriere , M. Weller , R. Stupp , et al., “Optimal Management of Elderly Patients With Glioblastoma,” Cancer Treatment Reviews 39, no. 4 (2013): 350–357, 10.1016/j.ctrv.2012.05.008. 22722053

[191] Y. Gong , R. Klein Wolterink , J. Wang , G. Bos , and W. Germeraad , “Chimeric Antigen Receptor Natural Killer (CAR‐NK) Cell Design and Engineering for Cancer Therapy,” Journal of Hematology & Oncology 14, no. 1 (2021): 73, 10.1186/s13045-021-01083-5. 33933160 PMC8088725

[192] B. M. Alexander and T. F. Cloughesy , “Adult Glioblastoma,” Journal of Clinical Oncology 35, no. 21 (2017): 2402–2409, 10.1200/jco.2017.73.0119. 28640706

[193] I. Preddy , K. Nandoliya , J. Miska , and A. U. Ahmed , “Checkpoint: Inspecting the Barriers in Glioblastoma Immunotherapies,” Seminars in Cancer Biology 86 (2022): 473–481, 10.1016/j.semcancer.2022.02.012. 35150865 PMC9363531

